# Postnatal colonization with human "infant-type" *Bifidobacterium* species alters behavior of adult gnotobiotic mice

**DOI:** 10.1371/journal.pone.0196510

**Published:** 2018-05-15

**Authors:** Berkley Luk, Surabi Veeraragavan, Melinda Engevik, Miriam Balderas, Angela Major, Jessica Runge, Ruth Ann Luna, James Versalovic

**Affiliations:** 1 Department of Pathology, Texas Children’s Hospital, Houston, Texas, United States of America; 2 Department of Pathology and Immunology, Baylor College of Medicine, Houston, Texas, United States of America; 3 Integrative Molecular and Biomedical Sciences Graduate Program, Baylor College of Medicine, Houston, Texas, United States of America; 4 Department of Molecular and Human Genetics, Baylor College of Medicine, Houston, Texas, United States of America; 5 Texas Children’s Microbiome Center, Texas Children’s Hospital, Houston, Texas, United States of America; Mayo Clinic Rochester, UNITED STATES

## Abstract

Accumulating studies have defined a role for the intestinal microbiota in modulation of host behavior. Research using gnotobiotic mice emphasizes that early microbial colonization with a complex microbiota (conventionalization) can rescue some of the behavioral abnormalities observed in mice that grow to adulthood completely devoid of bacteria (germ-free mice). However, the human infant and adult microbiomes vary greatly, and effects of the neonatal microbiome on neurodevelopment are currently not well understood. Microbe-mediated modulation of neural circuit patterning in the brain during neurodevelopment may have significant long-term implications that we are only beginning to appreciate. Modulation of the host central nervous system by the early-life microbiota is predicted to have pervasive and lasting effects on brain function and behavior. We sought to replicate this early microbe-host interaction by colonizing gnotobiotic mice at the neonatal stage with a simplified model of the human infant gut microbiota. This model consortium consisted of four “infant-type” *Bifidobacterium* species known to be commensal members of the human infant microbiota present in high abundance during postnatal development. Germ-free mice and mice neonatally-colonized with a complex, conventional murine microbiota were used for comparison. Motor and non-motor behaviors of the mice were tested at 6–7 weeks of age, and colonization patterns were characterized by 16S ribosomal RNA gene sequencing. Adult germ-free mice were observed to have abnormal memory, sociability, anxiety-like behaviors, and motor performance. Conventionalization at the neonatal stage rescued these behavioral abnormalities, and mice colonized with *Bifidobacterium* spp. also exhibited important behavioral differences relative to the germ-free controls. The ability of *Bifidobacterium* spp. to improve the recognition memory of both male and female germ-free mice was a prominent finding. Together, these data demonstrate that the early-life gut microbiome, and human “infant-type” *Bifidobacterium* species, affect adult behavior in a strongly sex-dependent manner, and can selectively recapitulate the results observed when mice are colonized with a complex microbiota.

## Introduction

A growing number of studies have established a role for the intestinal microbiota in the development of the central nervous system (CNS). This bi-directional communication between gut microbes and their host is commonly referred to as the ‘microbiota-gut—brain axis’ [1–5]. Early-life modulation of the microbiota-gut-brain axis is hypothesized to have significant effects which last into adulthood. Studies using gnotobiotic rodent models have provided key insights to the differences in behavior between adult germ-free (GF) or microbiota-depleted mice, and those with a complex murine microbiota [6–8]. Microbial colonization status affects stress responsivity, anxiety-like behavior, learning and memory, fear-recall, and sociability behaviors [9–18]. It has also been established that there is a critical window of development during which re-colonization of germ-free mice with a complex microbiota (conventionalization) can rescue the behavioral abnormalities observed in germ-free mice [11, 12, 19–21]. However, the infant microbiota is vastly different from the adult microbiota in both humans and mice, and early conventionalization with an adult microbiota may not sufficiently recapitulate effects of the neonatal microbiome on neurodevelopment [22, 23]. In the study described herein, we sought to determine how variations in neonatal colonization affect adult behavioral phenotypes using a gnotobiotic mouse model colonized at postnatal day 1 (P1) with a simplified model of the human infant microbiome.

*Bifidobacterium* species are gram-positive anaerobes, and are among the first groups to colonize the infant gut. In humans, *Bifidobacterium* species are detectable between 3–6 days after birth, and compose up to 80% of the neonatal intestinal microbiota by the end of the first week [24–27]. Microbial colonization during the first 2–6 months after birth coincides with the maturation of neural circuitry in the brain and the establishment of behavior patterns during this early period of postnatal development [3, 28]. Additionally, previous studies have demonstrated that “infant-type” *Bifidobacterium* species have neuromodulatory effects in adult rodent models [29–36]. *Bifidobacterium* species are therefore attractive models of early-life microbiota-gut-brain communication. Herein, we present data supporting the hypothesis that early *Bifidobacterium* colonization plays a role in neurodevelopment and behavioral patterning.

**Fig 1:**
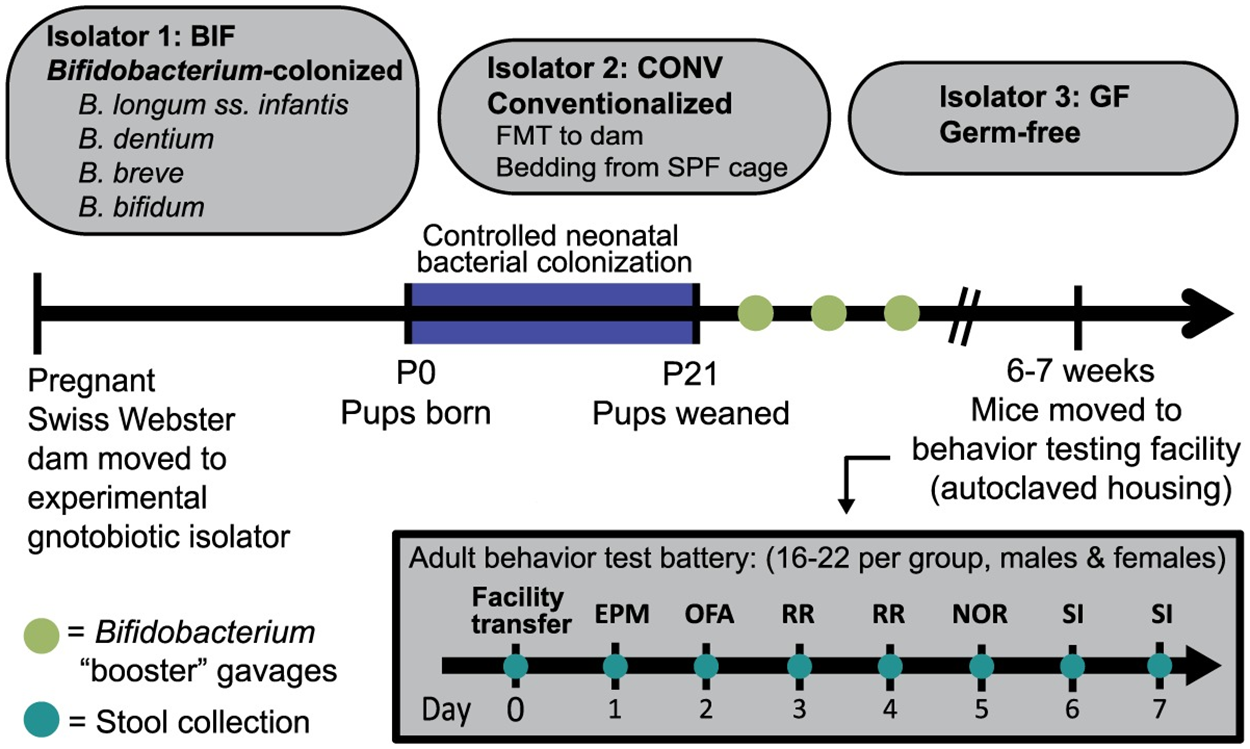
Neonatal colonization with a complex microbiota and a simplified model of the human infant microbiota. Controlled colonization of pups and dams in each gnotobiotic isolator occurred during the neonatal, P0 –P21 developmental window (P = postnatal day). Pups received oral gavages of either the *Bifidobacterium* treatment (BIF group), or sterile PBS (CONV and GF groups). The Fecal Microbiota Transplant (FMT) was delivered to the CONV dam on P1. After transfer out of the gnotobiotic isolators, the order of behavior testing performed in adult (6–7 week old) mice was: EPM = Elevated Plus Maze, OFA = Open Field Assay, RR = Rotarod, NOR = Novel Object Recognition, and SI = Social Interaction. See the Methods section and S1 Extended Methods for additional experimental detail. Results presented as mean ± SEM. GF = germ-free (n = 9m/13f), CONV = Conventionalized (n = 11m/8f), BIF = *Bifidobacterium*-colonized (n = 8m/9f).

Our simplified model was designed to mimic a human infant gut microbiota predominated by *Bifidobacterium*. We treated the mice starting at postnatal day 1 (P1) with four human-derived *Bifidobacterium* species known to be abundant in the intestine of healthy human infants (“infant-type”), yet are relatively diminished in adults [24, 37, 38]. These species included *Bifidobacterium longum* subsp. *infantis*, *Bifidobacterium breve*, *Bifidobacterium bifidum*, and *Bifidobacterium dentium*. Separate control groups included germ-free mice, which remained devoid of bacteria throughout development, and mice that were conventionalized at the neonate stage. Conventionalized mice were colonized with a complex murine microbiota which they acquired via natural routes of transmission from the dam and cage environment after birth. The mice matured to adulthood (6–7 weeks) in the gnotobiotic isolators, then underwent testing to assess a variety of behaviors (Fig 1). We also performed an in-depth characterization of the gut microbiota of each group of mice, and examined the dynamics of changes in these microbial communities after the mice were removed from the gnotobiotic isolators for behavioral testing. With this experimental manipulation of postnatal bacterial colonization, we aimed to better understand how the early-life microbiome affects long-term changes in animal behavior.

In agreement with results published by several independent groups, we observed both motor and non-motor behavioral abnormalities in germ-free mice relative to the conventionalized group [6, 12, 39, 40]. These previous studies have set a precedent for the consideration of behaviors as “normal” versus “abnormal”, with respect to microbial-colonization state. “Normal” behavior is defined here as that which would be seen in a healthy, wild-type mouse (with an unaltered, pathogen-free gut microbiome) relative to the “abnormal” behaviors typically observed in germ-free mice. In this study, the behaviors observed in the conventionalized mice are defined as “normal” based on the similarity of the group’s microbiome profile to that of the SPF (Specific Pathogen Free) donor microbiome profile (unaltered, healthy murine microbiota). We examined how the behavior of mice colonized with the *Bifidobacterium*-predominated model microbiota compared with behaviors observed in the conventionalized and germ-free groups. This strategy allowed us to determine whether this simplified model could rescue the behavioral deficits of the germ-free group and also provided insight as to the specific behavior domains affected by these “infant-type” *Bifidobacterium* species. Our data indicate that *Bifidobacterium* colonization mimics select effects of colonization with a complex murine microbiota, restoring aspects of normal anxiety-like behavior in females and significantly improving recognition memory in adult male and female mice.

## Materials and methods

### Bacterial strains, media, and culture conditions

*Bifidobacterium* species were selected as model microbes of the human infant gut microbiota, since they are predominant species in the human infant gut. These four strains are referred to throughout the text as “infant-type”. *Bifidobacterium dentium* ATCC 27678, *Bifidobacterium longum* subsp. *infantis* ATCC 15697, *Bifidobacterium breve* ATCC 15698, and *Bifidobacterium bifidum* ATCC 29521 were obtained from the American Type Culture Collection (ATCC). All four strains of bifidobacteria were cultured in de Man, Rogosa, and Sharpe (MRS) medium (BD, Sparks, MD, USA) and grown under anaerobic conditions (Anaerobe Systems, Morgan Hill, CA) at 37 °C.

### Gnotobiotic mouse models

Additional information regarding mouse models is detailed in S1 Extended Methods. Briefly, Swiss Webster Mice (outbred) were purchased from Taconic, and bred in the germ-free facility at Baylor College of Medicine to create an outbred colony of germ-free mice. At approximately 6–8 weeks of age, timed pregnancies were induced in adult female mice. Pregnant dams and their litters were maintained under germ-free or gnotobiotic conditions in separate flexible isolators fed with HEPA-filtered air. The day of birth was considered as P0. Mice were kept under filter-top cages with *ad libitum* access to irradiated food and water. All animals were housed under 12 hour light– 12 hour dark cycle. Animal care and experimental procedures were approved by the Institutional Animal Care and Use Committee (IACUC) at Baylor College of Medicine, Houston, TX, in accordance with all guidelines set forth by the U.S. National Institutes of Health (Baylor IACUC approved protocols #AN-6530 and #AN-6706).

### Colonization of germ-free mice

Additional information regarding colonization of mouse models is detailed in S1 Extended Methods. *Bifidobacterium* strains were grown in culture conditions as described (under anaerobic conditions at 37°C). Overnight bacterial cultures were combined in equal ratios based on OD_600_ readings (OD_600_ of ~1.0/species), pelleted, and re-suspended in sterile phosphate buffered saline (Gibco, Grand Island, NY, USA). This mixture of the four strains of *Bifidobacterium* used for the gavages is referred to throughout the text as the “*Bifidobacterium* treatment”. The dam was administered 0.2 mL of this treatment via oral gavage every other day post-partum and via drinking water. Pups were treated with 0.02 mL of the gavage mixture starting at P1, and every other day thereafter until P20. Dilution plating of the gavage mixture at several points during the experiment on MRS plates confirmed that the dams received approximately 1.1x10^10^ CFUs (colony forming units—i.e. viable bacterial cells) per gavage, and the pups received approximately 1.1x10^9^ CFUs. The pups were then weaned at P21 (2–5 mice per cage), and received a single gavage of the *Bifidobacterium* treatment once per week until behavior testing (13 total treatments).

Control germ-free mice received gavages of sterile PBS in the same manner as described above. Germ-free status was monitored over the course of the experiment by plating fresh fecal pellets from the dams on blood agar plates (Hardy Diagnostics, Santa Monica, CA, USA) and incubating both aerobically and anaerobically at 37°C. An absence of bacterial colonies on these plates 48 hours later was considered negative for bacterial contamination.

Conventionalized (CONV) litters were colonized by administering fecal matter derived from Specific Pathogen Free (SPF) donor mice to the dams via gavage and by adding SPF fecal matter to the cage environment housing the pups. A single cage of strain, age, and sex-matched (Swiss Webster, 6-week-old adult, female), SPF mice were used as feces donors, in order to maintain consistency throughout the experiment. Only the dams were given the gavage of fecal matter (on P1), and used bedding material from the cage of SPF donor mice was added to the cage with the dam and new pups (on P1 and P3). In this way, the pups were colonized via close interactions with the dam, and via environmental exposure to the fecal matter in the bedding. Consistent handling throughout development between the groups was maintained by administering gavages of sterile PBS to the conventionalized litters.

### Mouse handling and behavior testing

Additional information regarding colonization of mouse models is detailed in S1 Extended Methods. At 6–7 weeks of age, the mice were transferred into sterile cages and moved to the facility housing the behavioral testing equipment. The numbers of mice used in the behavior testing were as follows: Germ-free, n = 9 males and n = 13 females; *Bifidobacterium*-colonized, n = 8 males and n = 9 females; Conventionalized n = 11 males and n = 8 females. The mice were allowed to acclimate to the new environment for 24 hours after removal from the gnotobiotic isolators, after which behavioral testing commenced. We examined one assay per behavior domain, and limited the test battery to one week to minimize exposure to environmental sources of bacterial contamination to the best of our ability. The mice were housed in sterile cages and provided sterile food and water during the behavior testing. Handling was minimized, and all equipment was cleaned thoroughly with 70% ethanol between tests. All tests were run under dimmed lighting (70 lux) with white noise background (60 dB) to avoid distraction and minimize anxiety. All testing occurred between 1200–1900 hours and mice were allowed to habituate to the testing environment for 30 minutes prior to testing. After behavioral testing, mice were euthanized either by CO_2_ inhalation under controlled conditions via a Euthanex chamber or in cases where tissue was collected, cervical disarticulation under surgical plane of anesthesia. As required by institutional policies, secondary methods to ensure death were utilized, in addition to decapitation or bilateral opening of the thorax. All euthanization procedures were performed by research personnel approved on the Baylor College of Medicine IACUC protocol #AN-6706.

#### Elevated plus maze (EPM)

The mice were tested on a plus-shaped apparatus elevated above the floor. The apparatus consists of two open arms and two enclosed arms extending from a common center. The walls and floor of the apparatus are made of black Plexiglas. Each animal was placed in the center of the EPM facing the open arm and activity was monitored by an overhead camera for the duration of the 10-minute test. The ANY-Maze software program (Stoelting Co., Wood Dale, IL, USA) was used to track the position of the mouse in the elevated plus maze and record time spent in each area of the maze. Grooming and rearing were also scored by the researcher throughout the duration of the test using the ANY-maze software.

#### Open field (OF)

The animals were placed individually in the center of the VersaMax (Omnitech Electronics, Inc, Columbus, OH USA) open field apparatus (40x40 cm clear Plexiglas) and were monitored continually during the 30-minute test by the fully automated system (15 blocks of 2-minute intervals). Each monitor was equipped with horizontal (x,y) and vertical (z) photocell emitters and receptors that recorded horizontal movement and vertical activity respectively. The analyzer recorded beam break information and calculated multiple activity measures over the preset time period, including distance travelled, movement time, number of rears (vertical activity), and time spent rearing (vertical activity time). This test was performed in the absence of research personnel.

#### Rotarod (RR)

An accelerating rotarod (Ugo Basile, Gemonio VA, Italy) was used to evaluate maximal motor performance. The mice were placed initially on a static rod. The rod then accelerated from 5 rpm to 40 rpm during the course of the 5-minute test. The latency to fall off the rotating rod and/or grip the rod firmly and passively somersault around twice (consecutively) was measured and used to score motor performance and coordination. This test was performed as 8 trials spread over 2 days (4 trials per day). Mice were given a one-hour rest between trials.

#### Novel object recognition (NOR)

The novel object recognition test was used to assess learning and memory in the mice. The arena was surrounded on three sides with a white screen to limit additional spatial information. This test was performed in two stages and the mice were habituated to an empty test arena for 5 minutes immediately prior to both stages of the test. During the first 5-minute test stage (familiarization), the mouse was placed in the arena with two identical objects. Activity data was recorded by an overhead camera and was scored by the semi-automated ANY-maze system (Stoelting Co.). Activities directed towards the object, including sniffing, pawing, or climbing on the object were scored as interactions. Mice were placed in their home cages and given *ad libitum* access to food and water for 1 hour between test stages. During the second test stage, the mice were placed in an arena with one novel and one familiar object, and activity data and interaction time was scored. The objects used in this test differed by color, texture, and shape and have been previously validated for use in this test. Object placement was randomized for each test. Due to camera malfunction during a test, one germ-free male mouse was excluded from analysis of this test.

#### 3-Chamber test for sociability (SI)

A three-chambered apparatus was used to measure sociability in the mice. The apparatus was made of clear Plexiglas divided into three equally sized chambers and was surrounded on three sides with a white screen. The test consisted of two 10-minute tests, the first measuring baseline activity in the apparatus and the second measuring sociability. In the first habituation test, the mouse was placed in the center chamber and allowed to explore the entire apparatus with two empty cups in the chambers for 10 minutes. The mouse was then briefly confined to the center chamber with two clear Plexiglas panels. The left and right chambers and cups were quickly cleaned with ethanol, then the partner mouse or object was placed under the cups. The side of placement for the partner mouse for each test was randomized. The object used was made of black plastic blocks approximately 5 cm wide x 6 cm tall. The partner mice used in this experiment were age and sex-matched C57Bl/6J mice, which were trained under the cups for one hour per day, for two days prior to testing to reduce movement in the cups on test day. During testing, the mouse was allowed to freely explore the apparatus for the duration of the 10-minute test, and activity data was recorded by an overhead camera and the semi-automated ANY-maze system (Stoelting Co.). Activities directed towards the cups, including sniffing and pawing were also scored. The mouse was then transferred to a holding cage until all mice in the home cage were tested. Although this test is usually performed before Novel Object Recognition in a typical test battery, we chose to perform this test last in order to avoid the microbial exposure that may be expected to result from a direct social interaction with a conventionally housed mouse that might affect the results of the tests earlier in the battery.

### Fluorescence *in situ* hybridization

**Fig 2:**
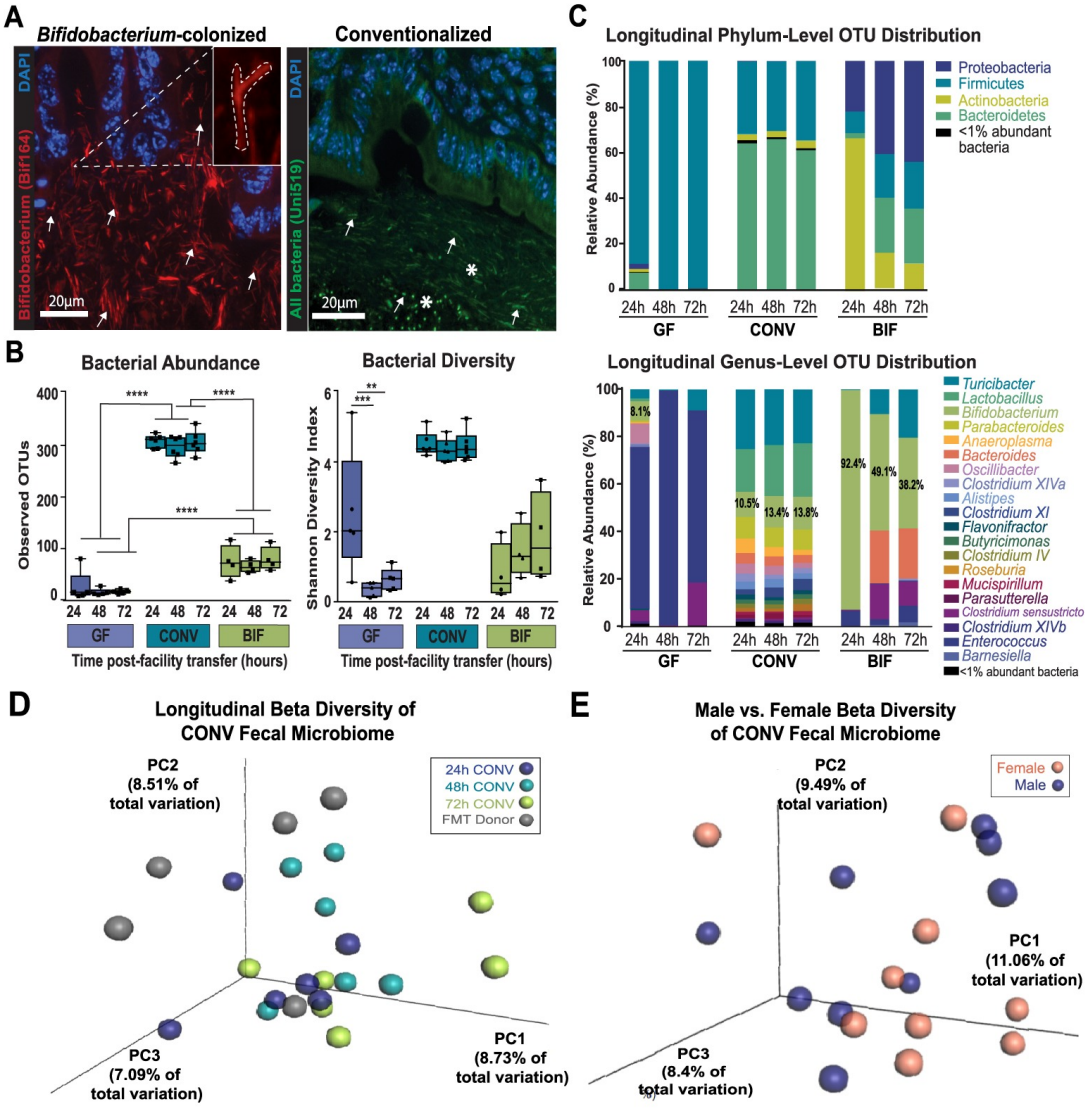
Longitudinal analysis of the gut microbiome in mice from each treatment group. **(A)** Representative micrographs from Carnoy’s-fixed and paraffin-embedded intestinal tissue. The left panel image is from the colon of a *Bifidobacterium*-colonized mouse and shows the result of an *in situ* hybridization with the Texas Red-labeled, *Bifidobacterium* genus-specific probe Bif164. The right panel shows colon tissue from a conventionalized mouse that was hybridized with the FITC-labeled universal bacterial probe, Uni519. Nuclei of host intestinal epithelial cells are counterstained in both images using DAPI. Inset in left panel demonstrates “bifid” morphology typical of *Bifidobacterium* species. White arrows indicate what was considered positive signal versus background in both panels, and white stars highlight the diversity of bacterial species present in the conventionalized mice as shown by the varying morphologies (rod vs. cocci) and localization. Images taken at 60x; scale bar = 20μm. **(B)** Alpha diversity of microbiota in each treatment group as measured by the Shannon Diversity Index (right panel) and Operational Taxonomic Unit (OTU) richness as measured by number of observed OTUs (left panel) at 24, 48, and 72 hours in each of the cohort groups. **(C)** Longitudinal relative abundance of OTUs. Phylum—level (Top panel) and Genus-level (Bottom panel) comparisons distributed by mouse group and timepoint post-transfer from gnotobiotic isolators. (D-E) Principal Coordinates Analyses (PCoA) showing **(D)** stability of the CONV fecal microbiome over time after transfer from gnotobiotic isolators, and **(E)** similarity between male and female fecal microbiome profiles in the conventionalized cohort. The percentage variation explained by each of the three primary principal factors is indicated on each axis. Coordinates representing individual samples are colored according to group, with distance to other coordinates indicating similarity/dissimilarity. (Data from 24h, 48h, and 72h timepoints in CONV group is combined in this analysis. Additional data detailing diversity by timepoint and sex in CONV group is shown in S1 Fig. Data also shown for other treatment groups parsed by timepoint in S1 Fig). Results presented as mean ± SEM. *p < 0.05, **p<0.01, ***p<0.001. (fecal microbiome analysis: n = 3m/3f per timepoint,t totaling n = 9m/9f per treatment) GF = germ-free, CONV = Conventionalized, BIF = *Bifidobacterium*-colonized.

A segment of intestinal tissue extending from the distal ileum to proximal colon was harvested from mice post mortem, after behavior testing was complete. The tissue was fixed in Carnoy’s fixative (American MasterTech Scientific, Inc., Lodi, CA, USA) at room temperature for 24 hours, then incubated in dry methanol prior to processing and embedding. 4 μm sections were mounted on glass slides, baked at 60°C for 1 hour, then de-paraffinized with xylene and dehydrated in series from 50% to 100% ethanol. The probes used were a previously validated [41], 5’ Texas Red-labelled *Bifidobacterium* genus specific probe (Bif164; 5’-CATCCGGCATTACCACCC-3’) or a 5’ Alexafluor 488-labelled universal bacterial probe (Uni519; 5’-GTATTACCGCGGCTGCTG-3’) targeted to the 164–181 bp or 519–536 bp regions of 16S rRNA, respectively (Integrated DNA Technologies, Coralville, IA, USA). The probe was hybridized to the samples by adding 15 μL of [2 μM] probe to each slide and placing in a 45°C hybridization chamber for 45 minutes. Nuclei were labeled with DAPI. Images were acquired using a Nikon Eclipse epifluorescence microscope. Representative images are shown in Fig 2, although intestinal sections from mice in each cage (total 5–6 per group) were utilized to confirm bacterial colonization.

### 16S rRNA sequencing and analysis

Fecal and cecal samples were used to assess the gut microbial community in adult mice after their removal from the gnotobiotic isolators. FMT preparations (feces in PBS) originating from SPF Swiss Webster female adult mice were also used to characterize the source microbial community. All samples were stored at -80°C until processing. Bacterial DNA was extracted using the MoBio Powersoil DNA Extraction Kit (Qiagen, Hilden, Germany), amplified, and sequenced by the Texas Children’s Microbiome Center. Briefly, 16S ribosomal RNA (rRNA) gene sequence libraries were generated targeting the V4 region of the 16S rRNA gene using the NEXTflex^™^ 16S V4 Amplicon-Seq Kit 2.0 (Bioo Scientific, Austin, TX) and sequenced on the Illumina MiSeq platform (Illumina, San Diego, CA, USA) with a median of 24,181 sequences generated per sample and a mean of 25,801 sequences generated per sample. The sequence libraries were host (mouse-based sequence) filtered using Bowtie2 [42]. Sequencing reads were quality filtered using LotuS [43] where sequences shorter than 200 base pairs, having average quality scores <20, containing ambiguous base calls, or including mismatches to barcode or sequencing primer were culled. After quality filtering and the removal of barcode and primer sequences (de-multiplexed reads), reads were pair-end stitched and remaining sequences were clustered into operational taxonomic units (OTUs) at a 97% similarity level using LotuS with Ribosomal Database Project Classifier [44]. Taxonomy was assigned using the Ribosomal Database Project Classifier trained on the SILVA v123 reference database [45]. Organisms potentially classified to the species level were based on individual OTUs of significance. OTUs failing to classify as bacteria at the kingdom level were removed prior to further analysis of the dataset. Bacterial community profiles were then characterized by the degree of diversity, richness, and relative abundance of the OTUs identified in each sample was evaluated using QIIME [46]. Output sequences were classified at the phylum and genus levels for comparisons within and across groups. OTUs that were classified under the genus *Bifidobacterium* that were further able to be classified into species level designations are detailed in S3 Fig.

### Statistical analysis

Data were analyzed for statistical significance using the GraphPad Prism software, Version 7 (La Jolla, CA, USA). For each behavior test, a two-way analysis of variance (ANOVA) using sex and treatment as factors was conducted to determine whether any significant interaction occurred. In cases where there was no significant interaction observed by two-way ANOVA, the results of the Bonferroni *post hoc* test are reported. In addition, the males and females were combined and a one-way ANOVA (or Kruskal-Wallis) test with appropriate *post hoc* test was used to evaluate statistical significance. In cases where we observed an interaction between sex and treatment, we considered the sexes separately and used a one-way ANOVA, followed by a Bonferroni Multiple Comparisons *post hoc* test to evaluate statistical significance. All data (sexes combined and separated) are shown in the figures, however statistical significance is only reported for the figures based on these parameters. The Rotarod data were analyzed via repeated measures ANOVA. Statistical significance was considered to be *p* <0.05. Non-parametric data were either log-transformed or tested using Kruskal-Wallis test where appropriate. All statistical data pertaining to behavioral testing are shown in S1 Table. Multivariate analyses of microbiome data including observed OTUs, Shannon Diversity Index, and relative abundances (percent frequency) were analyzed for statistical significance using the STAMP software package. Statistical significance was calculated using a two-tailed White’s non-parametric t-test for two-group comparisons and Kruskal-Wallis non-parametric ANOVA for multi-group comparisons. The Tukey-Kramer post-hoc test was further used to differentiate between groups when significance was calculated via the Kruskal-Wallis test, and all *p* values reported were Bonferroni corrected for multiple comparisons. Principal Coordinates analyses were performed in Graphpad, and PCoA plots were generated using EMPeror [47].

## Results

### Controlled neonatal colonization persists into adulthood

In this experimental paradigm, mice received the *Bifidobacterium* treatment via oral gavage every other day from P1 until P20, and then once per week until they were moved to the behavior testing facility (Fig 1). The conventionalized pups were also colonized during the pre-weaning period (P1-P20) via exposure to the dam and cage environment (conventionalization procedure detailed in S1 Extended Methods). Germ-free and conventionalized pups also received gavages of sterile PBS on alternating days until P20 to ensure all pups involved in later experiments were handled under the same conditions. Stool microbial communities were profiled post-behavior testing via sequencing of the V4 region of the 16S ribosomal RNA gene, and fluorescent *in situ* hybridization (FISH) was used to confirm intestinal localization of bacterial species present in *Bifidobacterium*-colonized and conventionalized mice post-mortem (Fig 2).

The sequencing analysis indicated that intestinal microbiome profiles were similar among conventionalized mice, regardless of sex or timepoint, as shown by the Principal Coordinates Anayses in Fig 2E and S1 Fig. Host sex explains a greater proportion of the variation in the germ-free and *Bifidobacterium*-colonized, versus conventionalized, groups (S1 Fig). The conventionalized group of mice was also observed to have a gut microbial community that was more rich and diverse than the germ-free or *Bifidobacterium*-colonized mice. This is demonstrated by the significant increase (*p*<0.0001) in Observed Operational Taxonomic Units (OTUs) between groups of conventionalized mice (CONV = 305 OTUs ± 4.469) and those that were germ-free or *Bifidobacterium*-colonized (GF = 81 OTUs ± 4.556, BIF = 118 OTUs ± 6.647) (Fig 2B). In addition, the diversity of the conventionalized mouse microbiome, as measured by the Shannon Diversity Index, indicates the presence of a rich variety of species relative to the other groups (Fig 2B). This rich and diverse community remained stable in the conventionalized group of mice until end of testing, 8 days after transfer from the gnotobiotic isolators (Fig 2C and S2 Fig). Using fluorescence in situ hybridization (FISH), a universal bacterial probe (Uni519) was used to confirm the presence of this complex microbial community in tissue sections obtained post-mortem. This staining demonstrates multiple bacterial morphologies colonizing the cecum and colon. These data are representative of a complex community of species wherein certain bacterial morphologies (rod-shaped) reside in the mucus layer close to the intestinal epithelium, while different bacterial morphologies (cocci) reside in the intestinal lumen. This FISH labeling indicates that the densest colonization was in the cecum of conventionalized mice, as shown in the representative image of Fig 2A (right panel). This supports the sequencing data which indicate that a diverse microbial community persisted in these mice throughout their lifespan.

The sequencing analysis additionally demonstrated that the taxonomic distribution of the conventionalized group of mice before, during, and after the time of behavior testing replicated the SPF donor fecal microbiome profile (Fig 2D and S2 Fig). The fecal microbiome of the conventionalized group had a significantly greater abundance of the genera *Turicibacter* (*p*<0.05) and *Anaerotruncus* (*p*<0.01), and differed (non-significantly) from the FMT donor in abundance of *Lactobacillus*, *Clostridium*, *and Bifidobacterium* (S2 Fig). Together, these data indicate that the method of colonization was successful, and that the microbiome established early in the conventionalized mice closely resembled that of a healthy SPF mouse. The conventionalized mouse microbiome profile is consistent with previous publications which demonstrate that the murine intestine is primarily dominated by members of the Bacteroidetes and Firmicutes phyla (S2 Fig). The sequencing data also indicate differences in relative abundance at the genus level in the cecum content relative to the fecal samples (S1 Fig). Overall, this community was stable and resistant to changes, even over the course of exposure to new microbial influences from the environment.

Sequencing analysis also confirmed that the gut microbiome of the *Bifidobacterium*-treated group of mice was predominated by the genus *Bifidobacterium* during the first 72 hours after transfer from the gnotobiotic isolators (24h = 92.4%, 48h = 49.1%, 72h = 38.2%) (Fig 2C and S3 Fig). A *Bifidobacterium* genus-specific probe (Bif164) was used to further assess *Bifidobacterium* colonization in the intestine of the *Bifidobacterium*-colonized adult mice (Fig 2A **left panel**). The *Bifidobacterium* genus-specific FISH labeling shows that *Bifidobacterium* colonization was most dense in the colon as shown in the representative image (though colonization was observed in the cecum as well). The bacterial morphologies are the typical ‘bifid’ shape, indicative of *Bifidobacterium* (inset in left panel of Fig 2A), and the bifidobacterial cells colonize the mucus layer lining the intestinal epithelium. Dense colonization was still observed in the cecum and colon at the end of the experiment, further supporting the conclusion that bifidobacterial colonization persisted to adulthood and was present during behavioral testing.

As shown in Fig 2C, both the *Bifidobacterium*-treated and germ-free adults acquired additional bacterial species from environmental sources after removal from the gnotobiotic isolators, however, there was not a significant increase in richness or diversity observed in these groups during the 24 to 72-hour timeframe (Fig 2B). In germ-free mice, *Clostridium* species dominate the microbial profile 24 hours after transfer, followed by gradual expansion by *Turicibacter* species. Similarly, *Bifidobacterium*-colonized mice are also rapidly re-populated by *Clostridium* and *Turicibacter* species, supplemented by *Bacteroides* and *Enterobacter* species. Overall, these data demonstrate that *Bifidobacterium* colonization promoted the repopulation of the gut with a diverse set of bacteria, while the germ-free gut was easily colonized by a few predominating bacterial species.

### *Bifidobacterium* colonization rescues the anxiolytic phenotype of germ-free females

**Fig 3:**
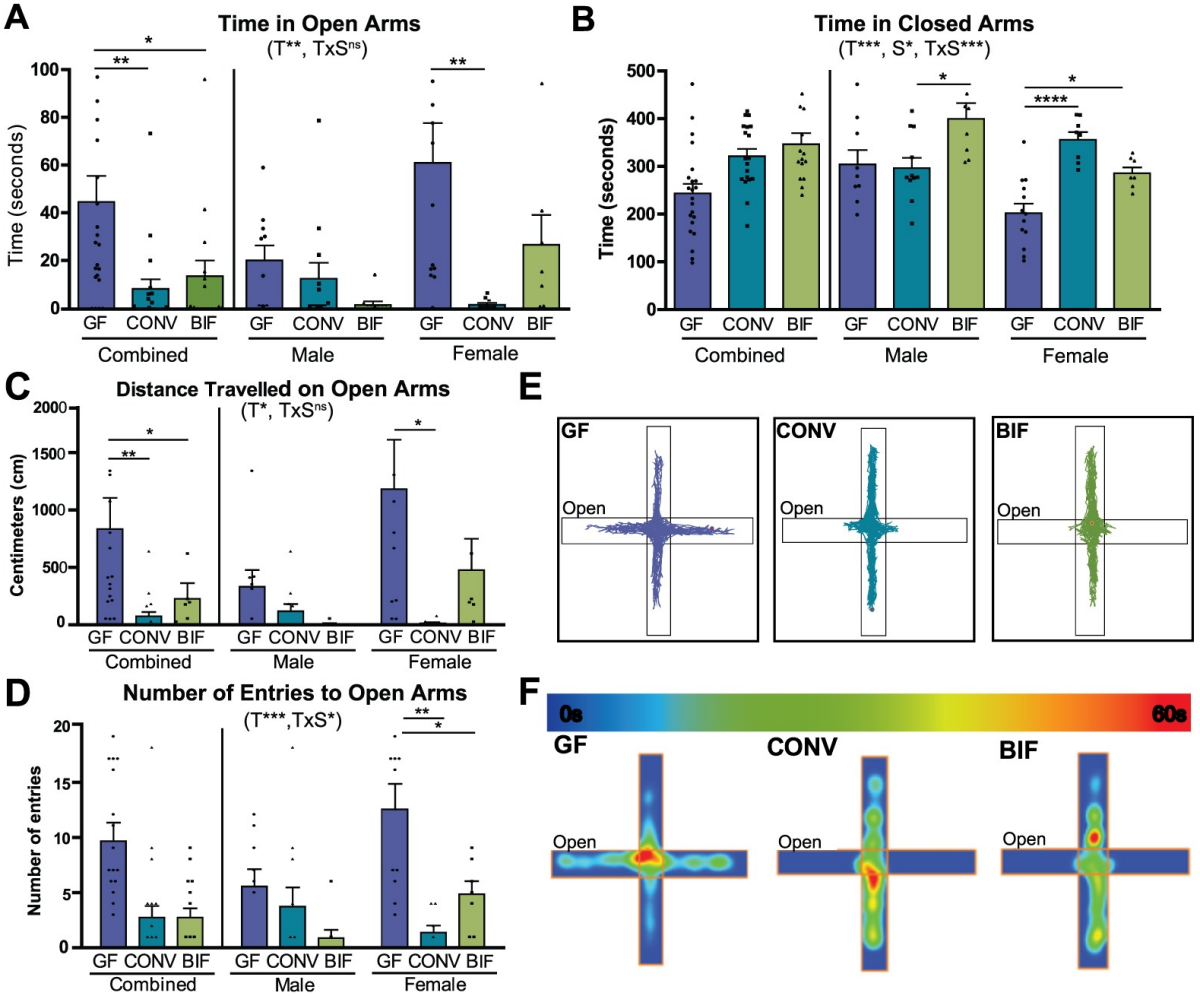
Anxiolytic behaviors of female germ-free mice in the elevated plus maze are normalized by *Bifidobacterium* colonization. **(A-B)** Bars show time (seconds) spent in the open and closed arms of the elevated plus maze by germ-free (GF), Conventionalized (CONV), and *Bifidobacterium*-colonized (BIF) mice during the 10-minute test session. **(C)** Bars represent total distance traveled (centimeters) on the open arms by each group of mice during the test session. **(D)** Bars represent total number of entries to the open arms of the maze during the test session **(E)** Representative track plots from female mice in each treatment group. The open arm of the maze is designated. **(F)** Representative heatmaps from female mice in each treatment group indicating how much time the animal spent (seconds) in different parts of the apparatus. The open arm is as designated. The scale of the heatmap ranges between 0 seconds (blue) and 60 seconds (red). All data (A-D) are presented as means ±SEM (n = 16–22 per treatment group, males and females). Significant treatment effects (T), sex effects (S), and interactions between treatment and sex (TxS) as determined by 2-way ANOVA are indicated under the title of each graph. Data are shown as sexes combined and sexes separated in the same graph for visualization purposes. Tests used to determine statistical significance notated in graphs are summarized in S1 Table. *p < 0.05, **p<0.01, ***p<0.001. Results presented as mean ± SEM. GF = germ-free (n = 9m/13f), CONV = Conventionalized (n = 11m/8f), BIF = *Bifidobacterium*-colonized (n = 8m/9f).

The elevated plus maze (EPM) is a well-established model for assessing anxiety-like behavior in mice [48, 49]. Mice that are more anxious spend more time in the darker closed arms, and less time exploring the more exposed open arms. Previous studies have established that most genetic backgrounds of germ-free mice (including Swiss Webster) display a reduced-anxiety, or anxiolytic, phenotype when compared with mice that develop with a complex microbiota [9–12, 17]. The treatment groups significantly differed in the amount of time spent in the open arms (*p* = 0.0077) and closed arms (*p* = 0.0008). When comparing effects of the variables in this test, significant interaction between sex and treatment was observed in the time spent in the closed arms (Fig 3A and 3B; *p* = 0.0009), indicating that the effect of the treatment was dependent on the sex of the mouse. Germ-free females displayed stereotypic anxiolytic behavior as demonstrated by the increased time spent in the open arms (*p*<0.01) and decreased time occupying the closed arms (*p*<0.0001) relative to conventionalized females (GF_open_ = 61.28s ± 16.70, CONV_open_ = 1.79s ± 0.86; GF_closed_ = 201.50s ± 20.06, CONV_closed_ = 355.2s ± 16.06). The behavior of the *Bifidobacterium*-colonized females paralleled the behavior of the conventionalized females as demonstrated by the decreased time spent in the open arms and increased time spent in the closed arms (*p*<0.05) relative to the germ-free females (BIF_open_ = 26.94s ± 12.51, BIF_closed_ = 285.3s ± 12.18). Conversely, in males, colonization with *Bifidobacterium* resulted in increased anxiety-like phenotypes relative to both the germ-free and conventionalized animals. These results indicate a sex-specific effect of intestinal microbiota on anxiety, which is more evident in males. While germ-free and conventionalized males did not significantly vary in average time spent in either arm, *Bifidobacterium*-colonized males spent less time exploring the open arms (BIF_open_ = 1.67s ± 1.54, GF_open_ = 20.38s ± 6.24, CONV_open_ = 12.67s ± 6.69) and significantly more time in the closed arms (BIF_males_ vs. CONV_males_ *p*<0.05; BIF_closed_ = 399.7s ± 33.14, GF_closed_ = 304.2s ± 30.05, CONV_closed_ = 296.6s ± 21.62

In addition to increased time spent in the open arms, female germ-free mice exhibited “riskier” behavior, as demonstrated by their increased exploration to the ends of the open arms (Fig 3C–3F). Significant differences between treatment groups were observed as total distances travelled on the open arms (Fig 3C; *F*(2,50) = 3.78, *p* = 0.0295) and numbers of entries to the open arms (Fig 3D; *F*(2,50) = 8.88, *p* = 0.0005). Germ-free females travelled significantly farther on the open arms than conventionalized mice (GF_females_ = 1183.0cm ± 430, CONV_females_ = 131.3cm ± 9.85), and were more likely to enter the open arms of the apparatus (GF_females_ = 12.54 entries ± 2.34, CONV_females_ = 1.375 entries ± 0.63).

Female mice colonized with the 4 *Bifidobacterium* species displayed behavior that more closely resembled the conventionalized group of mice as seen by significantly decreased distance travelled on the open arms (BIF_males_ = 6.75 cm ± 6.75, BIF_females_ = 475.3 cm ± 274.3) and fewer entries to the open arms relative to the germ-free females (BIF_males_ = 0.87 entries ± 0.74, BIF_females_ = 4.86 entries ± 1.184). These data support previously published literature suggesting that the reduced-anxiety phenotype of germ-free mice is reversible only if mice are colonized with intestinal bacteria early in life as no detectable behavioral deficits are observed when germ-free mice are colonized as adults [12, 49]. The data presented here further indicate that postnatal colonization with a small consortium of select *Bifidobacterium* species is sufficient to recapitulate a subset of the results observed when mice are colonized with a complex microbiota, however it must be emphasized that sex of the animal plays a role in determining the extent of the effect.

### Only colonization with a complex microbiota can rescue the hyper-locomotive phenotype of female germ-free mice

**Fig 4:**
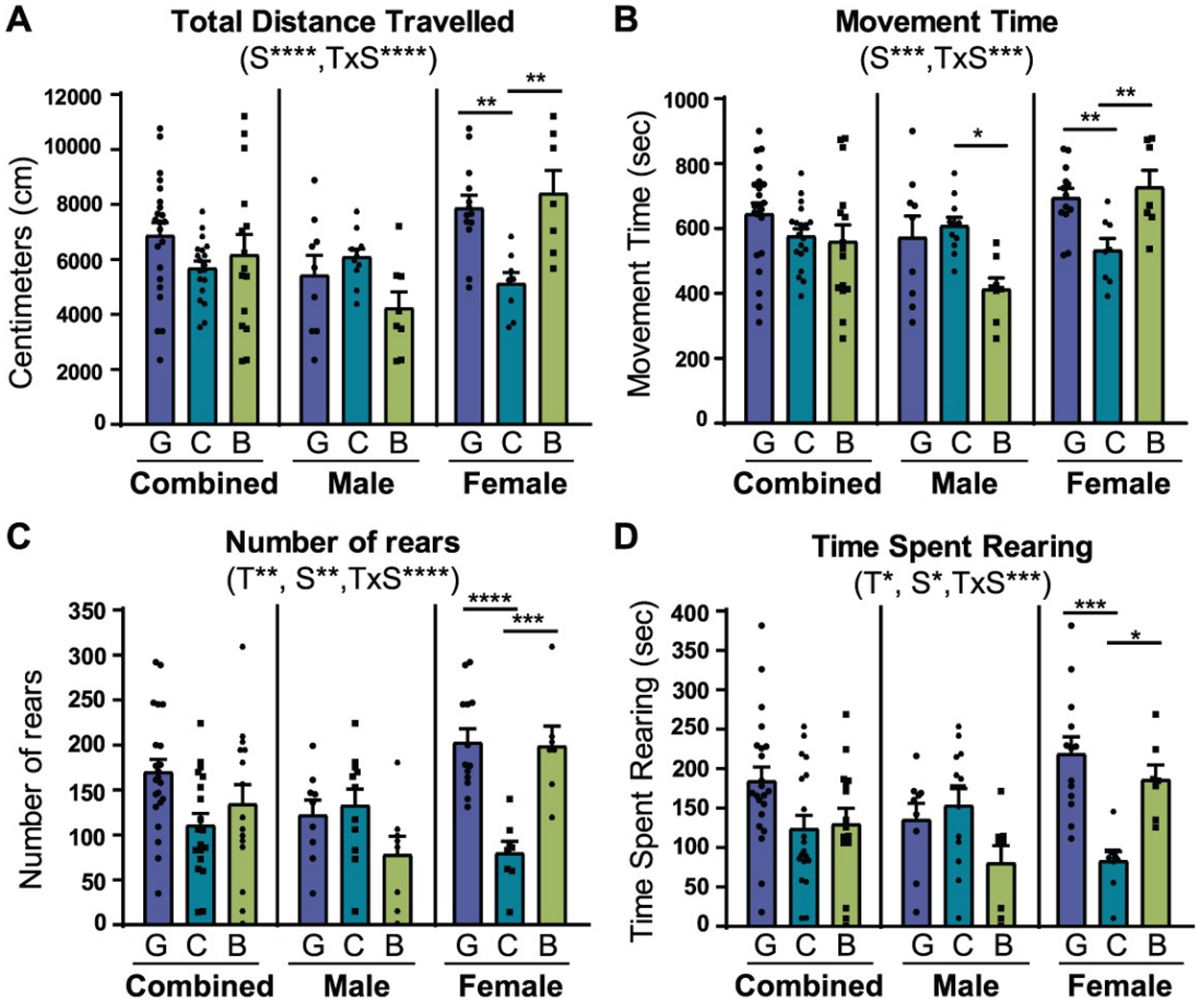
Hyper-locomotive phenotype of female germ-free mice is only rescued by complex microbiota. **(A)** Bars show mean (±SEM) cumulative distance travelled (centimeters) in the open field arena by germ-free (G), conventionalized (C), and *Bifidobacterium*-colonized (B) mice during the 30-minute test session. **(B)** Bars show mean (±SEM) cumulative time (seconds) that mice in each treatment group spent in active movement during the test session. **(C)** Bars represent mean rearing activity (average number of rears ±SEM) of each group of mice during the test session. **(D)** Bars represent average time spent rearing (seconds) during the test session. All data (A-D) are presented as means ±SEM. Significant treatment effects (T), sex effects (S), and interaction between treatment and sex (TxS) as determined by 2-way ANOVA are indicated under the title of each graph. Data are shown as sexes combined and sexes separated in the same graph for visualization purposes. Tests used to determine statistical significance notated in graphs are summarized in S1 Table. *p < 0.05, **p<0.01, ***p<0.001, ****p<0.0001. Results presented as mean ± SEM. GF = germ-free (n = 9m/13f), CONV = Conventionalized (n = 11m/8f), BIF = *Bifidobacterium*-colonized (n = 8m/9f).

The open field test is used to assess locomotive activity by recording an animal’s movements around an open arena [50]. Previous studies have shown that germ-free mice have increased spontaneous motor activity levels in this test [10, 12]. Our results support this data and also indicate that there is a significant interaction between treatment and sex in several parameters measured by this test: total distance (*p* = 0.0001), movement time (*p* = 0.0002), number of rears (*p*<0.0001), and time spent rearing (*p* = 0.0004). Germ-free females displayed a hyper-locomotive phenotype relative to conventionalized mice (Fig 4), as demonstrated by the significant increase in total distance travelled (Fig 4A, GF_females_ = 7870 cm ± 464.3, CONV_females_ = 5125 cm ± 404.8, *p*<0.01), movement time (Fig 4B, GF_females_ = 695.7 sec ± 28.27, CONV_females_ = 533.7 sec ± 36.0, *p*<0.05), number of rears (Fig 4C, GF_females_ = 202.8 ± 15.15, CONV_females_ = 79.88 ± 12.98, *p*<0.0001), and time spent rearing (Fig 4D, GF_females_ = 218.8 sec ± 21.58, CONV_females_ = 82.23 sec ± 13.67, *p*<0.001). However, these behaviors failed to be rescued by the neonatal *Bifidobacterium* treatment, as BIF mice tended to retain their germ-free hyper-locomotive phenotype (Fig 4A, BIF_females_ = 8405 cm ± 837.7; Fig 4B, BIF_females_ = 727.7 sec ± 51.89; Fig 4C, BIF_females_ = 198.7 ± 22.09; Fig 4D, BIF_females_ = 186.3 sec ± 18.64).

In contrast, we observed that male mice treated with *Bifidobacterium* had decreased activity relative to both GF and CONV mice as shown by the significantly shorter time spent in active movement during the test (Fig 4B, GF_males_ = 573.0 sec ± 65.95, CONV_males_ = 609.0 sec ± 25.62, BIF_males_ = 413.6 sec ± 33.94, *p*<0.05), and trended towards decreased activity in other behavioral parameters assessed in the open field test. These data suggest that a complex interaction exists between sex and colonization status, in which *Bifidobacterium* treatment may have a greater effect on locomotion in females.

### Germ-free males have decreased motor performance that can be rescued by early *Bifidobacterium* colonization

**Fig 5:**
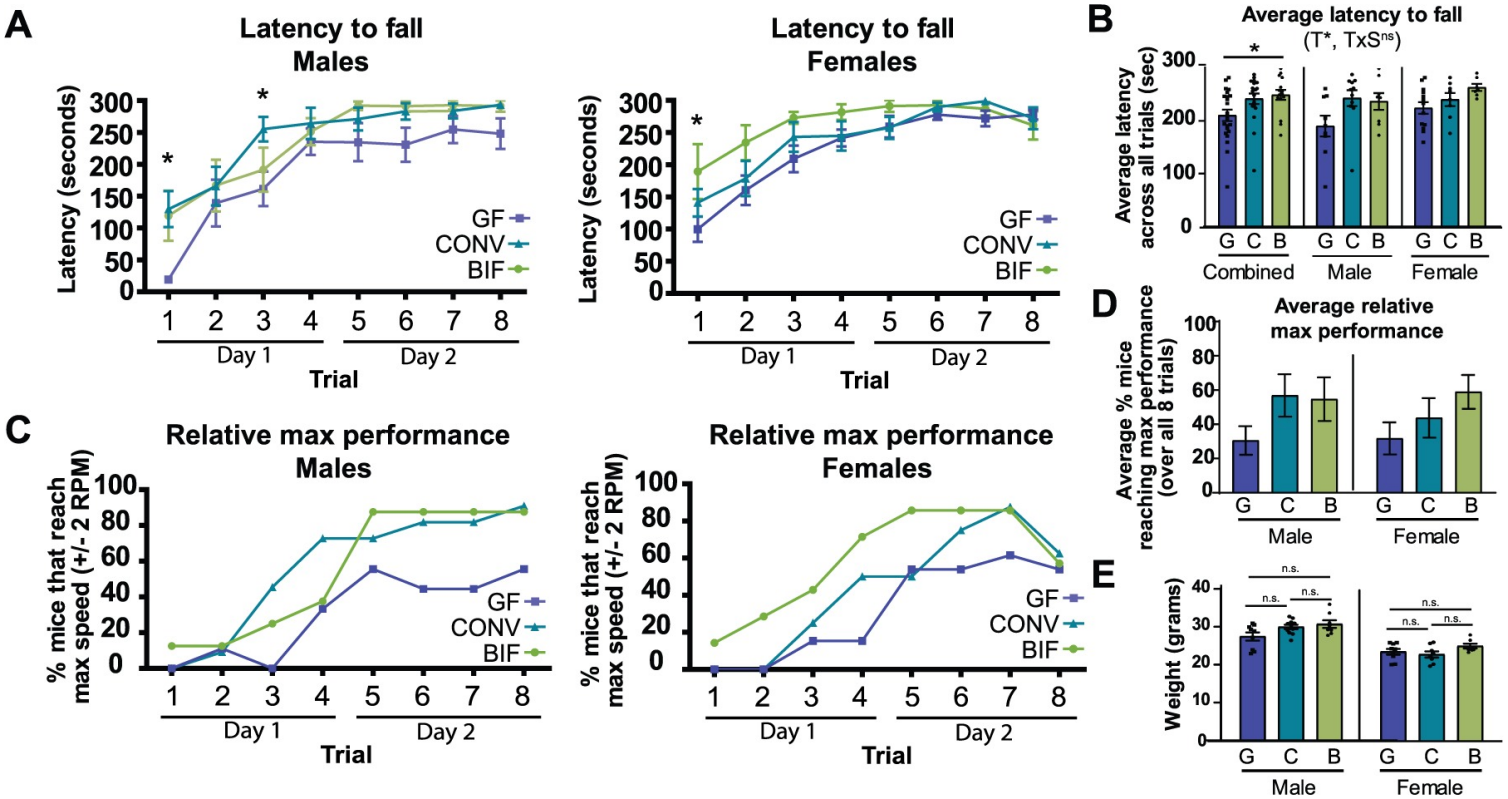
*Bifidobacterium* colonization improves motor performance defect of germ-free mice. **(A)** Plots show the mean latency (±SEM) to fall from the rotating rod over the course of 8 trials (4 trials on day 1, and 4 trials and day 2). Males and females are plotted separately **(B)** Average latency to fall from the rod (±SEM) averaged over all 8 trials for each group of mice. **(C)** Plots represent the relative maximum performance of males and females in each treatment group (percentage of mice that were capable of reaching maximum speeds (40 RPM ± 2) on the Rotarod apparatus during each trial). **(D)** Plot represents the average percentage of mice in each group that reached peak performance over all 8 trials. **(E)** Weight of male and female mice in each treatment group at time of testing. All data (A-E) are presented as means ± SEM. Significant treatment effects (T), sex effects (S), and interaction between treatment and sex (TxS) as determined by 2-way ANOVA are indicated under the title of each graph. Data are shown as sexes combined and sexes separated in the same graph for visualization purposes. Tests used to determine statistical significance notated in graphs are summarized in S1 Table. *p < 0.05 (GF (G) = germ-free, ■ (n = 9m/13f); CONV (C) = conventionalized, ▲ (n = 11m/8f); BIF (B) = *Bifidobacterium*-colonized, ● (n = 8m/9f)).

The Rotarod is a sensitive test designed specifically to allow for the automated measurement of neurological deficits in rodents [50]. The accelerating rod paradigm is commonly used to examine motor performance and coordination, and was performed here as a series of 8 trials spread over 2 days. Mice were placed on a notched beam that rotated about its axis and smoothly accelerated from 5 to 40 rpm throughout the 5-minute test period, during which time the animal must walk forward to remain upright and not fall off [50, 51]. Motor performance is measured by the latency to fall from the beam or to cartwheel around the beam twice, as well as the speed at which the fall occurred. When sexes are considered together, treatment has a significant effect on performance (*p* = 0.0101) with germ-free mice having a significant defect in motor performance relative to *Bifidobacterium*-treated mice as denoted by their decreased average latency to fall across all trials (Fig 5A; *p*<0.05). Considering the sexes separately, male germ-free mice displayed decreased motor performance relative to conventionalized males (Fig 5B; GF_males_ = 190.4 sec ± 19.70, CONV_males_ = 243.3 sec ± 15.17). *Bifidobacterium*-treated male mice displayed a trend towards improved motor performance, and performed similarly to the conventionalized mice as shown by increased time spent on the rod (BIF_males_ = 237.0 sec ± 15.86). This effect is especially apparent in the first trial, during which the germ-free males performed significantly poorer than both conventionalized and *Bifidobacterium*-treated males, possibly indicating a deficit in motor coordination (Fig 5A; *p*<0.05). In addition, 64% of conventionalized males reached peak test performance (maximum speeds of 38–40 rpm without falling) by trial 4, and 91% achieved this by trial 8 (Fig 5C). In contrast, only 33% of germ-free males reached the maximum speeds by trial 4, and only 55% by trial 8. The *Bifidobacterium*-treated males were observed to behave more like the conventionalized males in this regard, with 87.5% of the mice able to reach maximum speed by the 8^th^ trial. In Fig 5D, the plots show the average percentage of mice in each group that reach peak performance over the course of all 8 trials. This data demonstrates a trend in which fewer germ-free mice (both males and females) reach peak performance relative to the colonized groups. A potential confounding variable is the weight of the animal, as heavier mice are known to exhibit decreased performance in this test relative to light mice. However, in our study, the *Bifidobacterium*-treated and conventionalized male mice displayed heightened motor performance even though their weights did not vary significantly from their germ-free counterparts (Fig 5E).

Germ-free females were also observed to respond differently in the Rotarod test based on colonization status. *Bifidobacterium*-treated females showed a trend towards increased motor performance relative to both germ-free and conventionalized females, as demonstrated by increased average latency to fall from the rod over all trials (Fig 5B; GF_females_ = 224.7 sec ± 10.97, CONV_females_ = 240.6 sec ± 12.50, BIF_females_ = 263.7 sec ± 6.66). This effect is likewise most apparent in the early trials, especially trial 1, in which germ-free females displayed a significant deficit in motor performance relative to *Bifidobacterium*-treated females (Fig 5A, *p*<0.05). Similar to the males, the female germ-free mice displayed an attenuated ability to maintain coordinated forward motion at the higher speeds of this test. While 71% of *Bifidobacterium*-treated and 50% of conventionalized females were able to reach max speeds by trial 4, only 15% of germ-free females achieved this speed (Fig 5C). All female groups of mice displayed exhaustion by trial 8 as denoted by the decreased percentage of mice able to maintain high speeds. However, during trial 7, 85% of *Bifidobacterium*-treated and 87% of conventionalized females reached maximum speeds, while only 61% of germ-free females reached these speeds. This difference cannot be attributed to weight, as all groups of mice were within a narrow weight range (Fig 5E). This is the first in-depth description of how germ-free mice perform in the Rotarod test. Collectively, these data indicate that germ-free mice display motor performance deficiencies, which can be corrected by neonatal conventionalization and partially rescued with a select group of *Bifidobacterium* species applied early in life.

### Neonatal *Bifidobacterium* colonization facilitates development of functional recognition memory in both sexes

The novel object recognition test is a highly validated test for recognition memory and has also been described as a valuable measure of cognition in rodents [52]. If memory is functioning normally, the mouse is expected to spend more time exploring the novel object than it does exploring the familiar object, due to the animal’s natural propensity for novelty. This pattern can be measured as the recognition index, or the time spent investigating the novel object relative to the total time investigating both objects. In this test, it was observed that treatment had an extremely significant effect on the ability of the mice to recognize the novel object (*p*<0.0001).

**Fig 6:**
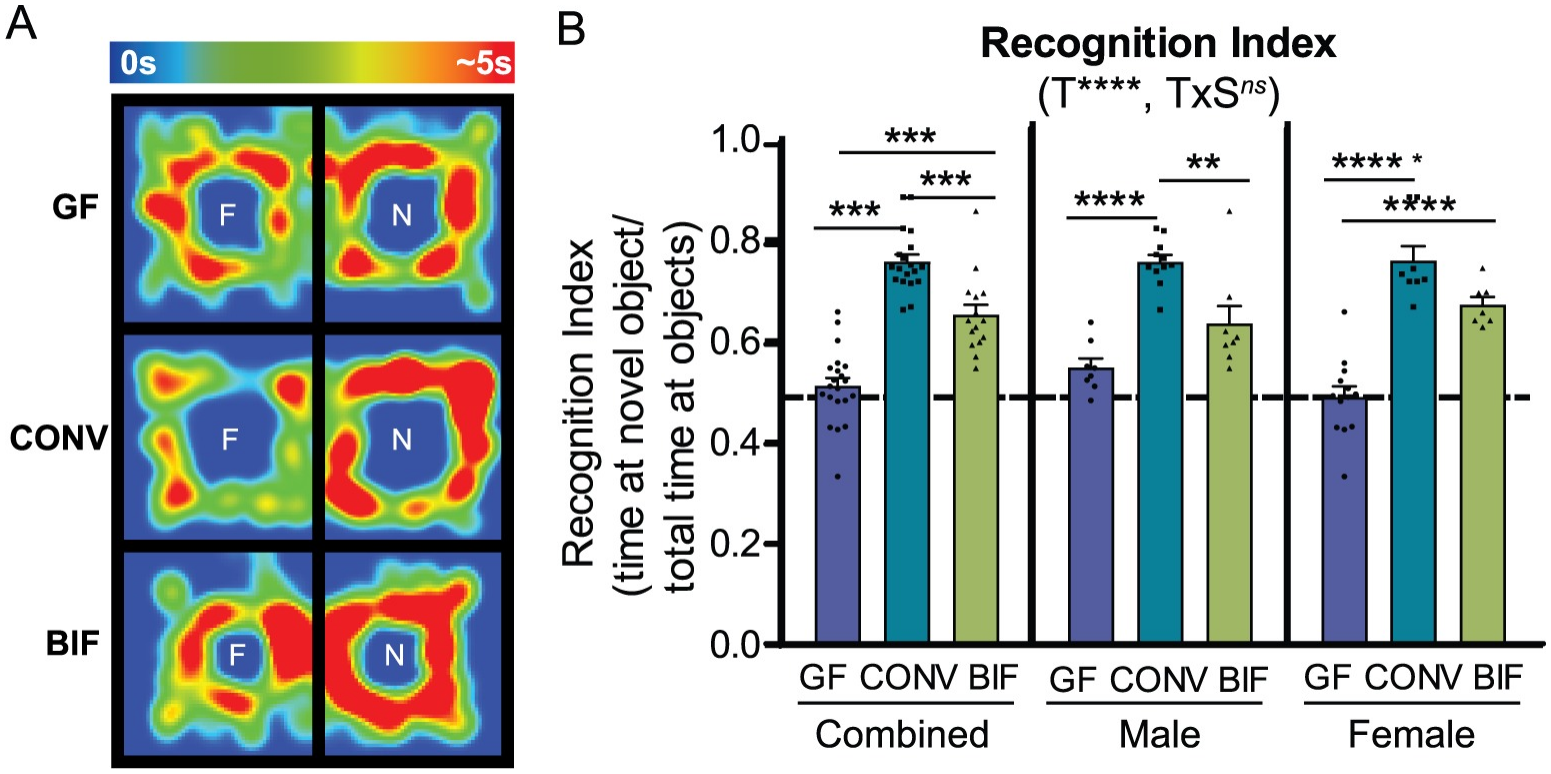
Neonatal *Bifidobacterium* colonization restores functional recognition memory in both males and females. **(A)** Representative heatmaps from female mice in each treatment group demonstrating time spent (seconds) around each object in the arena (F = familiar object, N = novel object). The scale of the heatmap ranges between 0 seconds (blue) and 5 seconds (red). **(B)** Bars represent the Recognition Index (time spent interacting with novel object / total time spent interacting with objects). Significant treatment effects (T), sex effects (S), and interactions between treatment and sex (TxS) as determined by 2-way ANOVA are indicated under the title of each graph. Data are shown as sexes combined and sexes separated in the same graph for visualization purposes. Tests used to determine statistical significance notated in graphs are summarized in S1 Table. *p < 0.05, **p<0.01, ***p<0.001, ****p<0.0001 Results presented as mean ± SEM. GF = germ-free (n = 9m/13f), CONV = Conventionalized (n = 11m/8f), BIF = *Bifidobacterium*-colonized (n = 8m/9f).

Both male and female germ-free mice spent approximately equal time exploring the novel and the familiar objects, indicating a memory deficit (Fig 6A and 6B; Recognition Index GF_males_ = 0.55 ± 0.02, GF_females_ = 0.49 ± 0.02). In contrast, both male and female conventionalized mice displayed a significantly improved recognition index (CONV_males_ = 0.76 ± 0.01, CONV_females_ = 0.77 ± 0.03), indicating recognition of the familiar object and therefore preference in exploring the novel object. Neonatal colonization with the defined set of *Bifidobacterium* species was also sufficient to restore the expected behavioral phenotype. *Bifidobacterium*-treated mice (both males and females) also displayed significantly improved recognition memory relative to germ-free mice (BIF_males_ = 0.64 ± 0.03, BIF_females_ = 0.68 ± 0.02). Together, these data suggest that the presence of either a complex gut microbiota or select commensal bifidobacterial species early in life can significantly improve functional recognition memory in adults.

### Neonatal colonization with a complex microbiota but not *Bifidobacterium* rescues the impaired sociability observed in adult germ-free females

**Fig 7:**
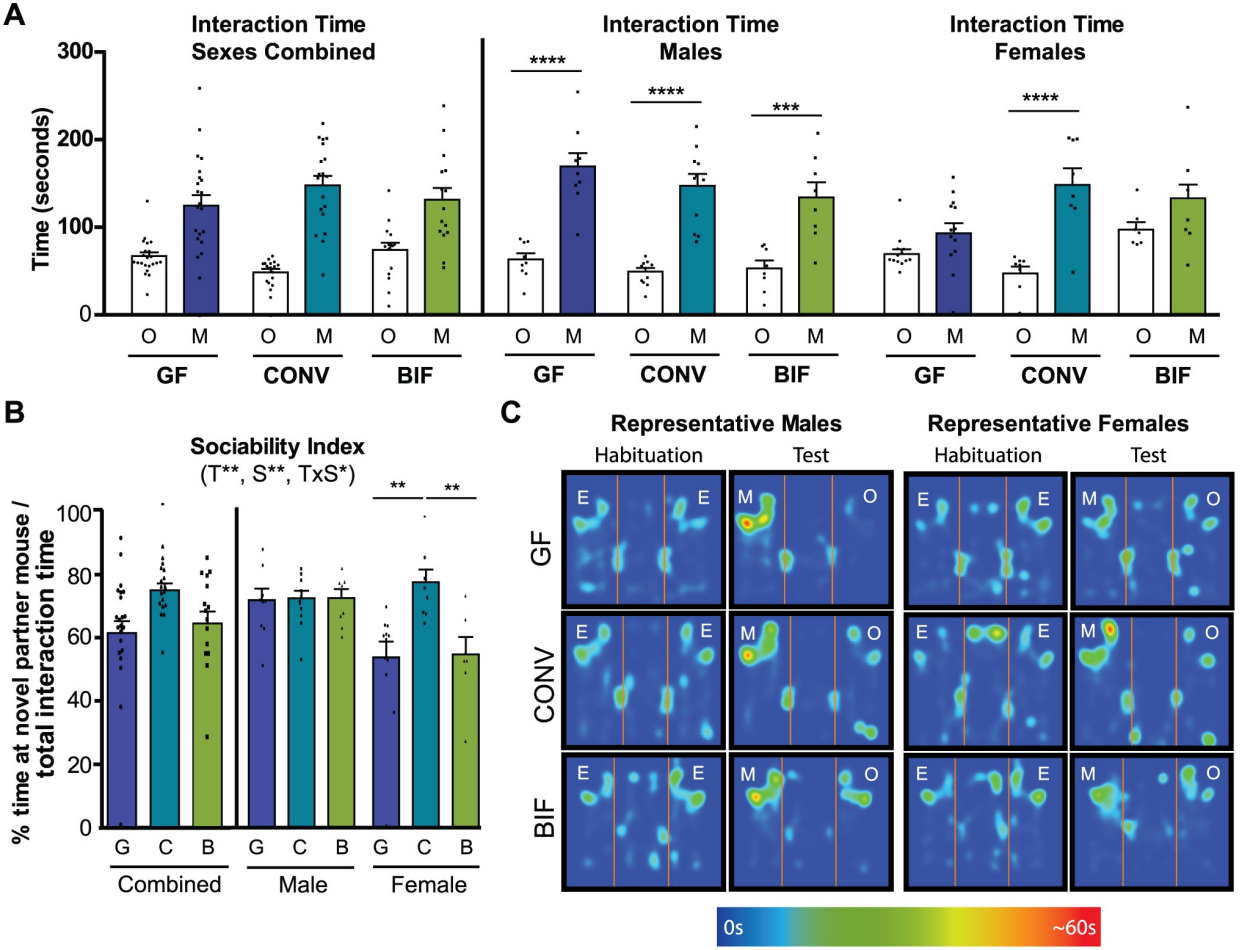
Sociability impairments of germ-free mice are rescued by colonization with a complex microbiota. **(A)** Bars show total time (seconds) that the test subject spent interacting with either the novel object (O) or novel partner mouse (M) cups during the 10-minute test session. Males and females are presented separately (combined sexes not shown) **(B)** Bars represent Sociability Index (% time spent interacting with the mouse cup/total time interacting with cups) by each group of mice during the test session. **(C)** Representative heatmaps from male and female mice in each treatment group demonstrating time spent interacting with empty cups (E) during the habituation trial, and interaction time around the object (O) or mouse (M) cups during the test trial. The scale of the heatmap ranges between 0 seconds (blue) and 60 seconds (red). Significant treatment effects (T), sex effects (S), and interactions between treatment and sex (TxS) as determined by 2-way ANOVA are indicated under the title of each graph. Data are shown as sexes combined and sexes separated in the same graph for visualization purposes. Tests used to determine statistical significance notated in graphs are summarized in S1 Table. *p < 0.05, **p<0.01, ***p<0.001, ****p<0.0001. Results presented as mean ± SEM. GF = germ-free (n = 9m/13f), CONV = Conventionalized (n = 11m/8f), BIF = *Bifidobacterium*-colonized (n = 8m/9f).

The three-chamber paradigm known as Crawley’s sociability test is a widely-employed test used to assess defects in sociability [53]. Sociability in this test is defined as the propensity to spend time with another mouse, as compared to time spent with a novel object in an identical chamber. This behavior can be quantified as the “sociability index”, or the time spent interacting with either the mouse cup or object cup as a percentage of total time spent at either cup combined. We observed that germ-free females, but not males, exhibited a deficit in sociability relative to conventionalized mice. All male mice preferred to spend significantly more time interacting with the novel mouse rather than the object regardless of treatment (Fig 7A; GF p<0.0001, CONV p<0.0001, BIF p<0.001; GF_object_ = 62.98 sec ± 6.84, GF_mouse_ = 168.8 sec ± 15.24, CONV_object_ = 48.90 sec ± 4.22, CONV_mouse_ = 146.9 sec ± 13.44, BIF_object_ = 53.13 sec ± 8.96, BIF_mouse_ = 134.1 sec ± 17.30). This behavior is reflected in the male sociability indices in which no significant differences were observed. Male mice in all three treatment groups spent approximately 72% of the total interaction time engaging with the novel partner mouse (Fig 7B). These results indicate that, in males, sociability was not affected by early life microbial colonization (conventionalization or *Bifidobacterium* treatment).

Conversely, female behavior significantly varied based on microbial colonization status (Fig 7A–7C). Conventionalized females spent significantly more time interacting with the novel mouse partner cup than the object cup, which is the expected social behavior for a healthy Swiss Webster mouse. Relative to conventionalized females, germ-free females spent significantly less time interacting with the novel partner mouse, indicating a sociability deficit. In comparison, *Bifidobacterium*-treated females showed a trend towards increased time spent engaging with the novel partner, but statistically, the *Bifidobacterium*-treatment was not able to “rescue” the sociability deficit observed in germ-free mice (Fig 7A; CONV p<0.0001; GF_object_ = 69.28 sec ± 5.61, GF_mouse_ = 93.09 sec ± 11.64, CONV_object_ = 46.98 sec ± 7.65, CONV_mouse_ = 148.0 sec ± 18.87, BIF_object_ = 96.89 sec ± 8.39, BIF_mouse_ = 127.2 sec ± 22.52). This finding is also reflected in the significant difference in female sociability indices between treatment groups (Fig 7B; GF vs. CONV p<0.01, BIF vs. CONV p<0.01). Conventionalized females spent on average 77.6% of time interacting with the novel mouse whereas Germ-free and *Bifidobacterium*-treated female mice spent on average 53.9% and 54.7% of time in social contact respectively. Overall, these data describe a strong sex-dependent effect of the microbiota on sociability, in which only female germ-free mice exhibited a sociability deficit relative to conventionalized mice. In addition, this deficit in females was not able to be fully rescued by early colonization with the simplified, *Bifidobacterium*-predominated microbiota. These data further highlight the sex- and microbiome-based specificity of microbiome-gut-brain interactions.

## Discussion

Microbiome-gut-brain axis communication begins in early life, and the composition of the initial gut microbial community and the resulting hologenome (sum of genetic information of a host and its microbiome) is predicted to have varying effects on these multisystem interactions. This study utilized gnotobiotic mice, colonized as neonates with a simplified model of the human infant gut microbiome to examine behavioral effects in adults. While previous studies have shown that members of the microbiota are capable of modulating host behavior, our data suggest intriguing complexity with respect to sex-dependent interactions occurring between microbiome and host. Our data also indicate that the presence of *Bifidobacterium*, has significant long-term impact on host behaviors, including mammalian memory.

Previous studies have documented behavioral differences in germ-free mice relative to mice colonized with a complex microbiota (SPF or conventionalized), but the study presented herein also focuses on how colonization with a limited consortium of four “infant-type” *Bifidobacterium* species affects adult behavior. We compare the behavioral outcomes of this bifidobacterial colonization with the outcomes observed when the mice have a conventional, complex microbiota. *Bifidobacterium* spp. colonize the infant GI tract during a critical window of postnatal human neurodevelopment. Recent studies have begun to define particular roles for *Bifidobacterium* in the microbiome-gut-brain axis. Research has shown that colonization with various *Bifidobacterium* species can beneficially impact stress responsivity, anxiety-like behaviors, and maternal separation-induced depressive-like behaviors in adult rodents [33, 35, 36, 54, 55]. Previous data also suggests that host behavioral responses to microbial manipulation are dependent on which specific *Bifidobacterium* species or strain is employed [30, 31, 36, 55]. Savignac *et*. *al*. demonstrated *Bifidobacterium* species-dependent reductions in host anxiety-like, depressive-like, and stress behaviors, as well as improvements in cognitive behaviors such as recognition memory and learning [30, 31]. The data presented herein support these findings and further highlight the importance of *Bifidobacterium* species and their ability to modulate select host behaviors. These data demonstrate that early colonization with *Bifidobacterium* species affects adult behavior in a sex-dependent manner and can selectively recapitulate the results observed when mice are colonized with a complex microbiota. We hypothesize that these species are excellent model microbes for studies of gut-brain crosstalk, and that by affecting circuit formation and neural patterning early in life, specific gut microbes are impacting downstream adult behavioral phenotypes.

A key result observed in this study was the sex-dependent interaction between microbial-colonization status of the gut and host behavior. A limited number of studies have previously reported potential sex-dependent interactions between microbe and host. These studies have shown male-specific behavioral effects in germ-free mice as well as neurochemical changes in the brain [10, 11, 14]. Our in-depth description of germ-free, gnotobiotic, and conventionalized mouse behavior, and the associated microbiome signatures helps establish how the presence (or absence) of certain members of the early-life gut microbiota may have different consequences depending on sex of the mammal. In our test for anxiety-like behavior for example, both male and female germ-free mice respond similarly relative to conventionalized mice, but *Bifidobacterium* colonization only restores function in females. Jašarević *et*. *al*. has suggested that these sex-dependent differences may originate from sexually dimorphic compositions of the gut microbiome in response to environmental factors throughout the mammalian lifespan [56]. Early-life variations in microbial communities may originate from differences in prenatal transport and uptake of maternal microbe-derived metabolites [56]. However, in the studies presented here, all dams were originally germ-free; therefore, the behavioral effects observed are more likely due to postnatal host-microbe interactions rather than prenatal exposure to microbial metabolites. Since no significant differences in microbiota composition were observed between conventionalized males and females, we hypothesize that the sex-dependent behaviors observed were the result of either a sex-specific effect of *Bifidobacterium* colonization, or that another indirect interaction between the microbiota and host physiology may be the cause. Sex-specific differences in the composition and variability of gut microbiome start to emerge during puberty and are driven by sex hormones such as androgens and estrogens [56, 57]. Consequences of microbial colonization may include sex-specific differences in neurotransmitter concentrations and gene expression in various regions of the brain, which may contribute to the sex-based differences observed [11, 58]. The presence of one species, or variations in species-level relative abundance between females and males may also be sufficient to drive behavioral differences. These studies highlight the importance of investigating both sexes in future studies regarding the gut microbiome and effects on the brain in development and adulthood.

Germ-free mice display abnormal behavioral phenotypes relative to mice that are born into a conventional environment (referred to as SPF), or those that are conventionalized early in life (born germ-free then colonized with the microbial component derived from SPF fecal matter, referred to as CONV) [6]. Multiple independent investigations have demonstrated the association between germ-free status and a reduction in anxiety-like behaviors [10–13]. The absence of gut microbiota has varying effects on anxiety-like behaviors based on the host sex and genetic background. Maturation in a germ-free state however can also exacerbate the anxious phenotype of certain mouse strains [10]. We demonstrate that germ-free Swiss Webster mice, females in particular, display a less-anxious phenotype and increased risk-taking behaviors. The results presented here concur with that previously published by Neufeld *et*. *al*., in which they demonstrate a decreased anxiety-like phenotype in female germ-free Swiss Webster mice as measured using the elevated plus maze [13]. Here we show that in females, both neonatal conventionalization and *Bifidobacterium*-colonization normalize these anxiety-like and risk-taking behaviors, further supporting the idea that early colonization is key to being able to rescue certain aspects of behavior. Other published studies have demonstrated that germ-free male Swiss Webster mice also display decreased anxiety-like behavior (relative to SPF mice) [10, 11]. However, in these studies the open field test and the light dark box were used to assess anxiety rather than the elevated plus maze, which precludes our ability to make direct comparisons to their results. Our results indicate that either *Bifidobacterium* or the conventional microbiota affects the anxiety-like behavior of males in a different way than females, re-emphasizing the importance of sex and host physiology in the complex interplay between microbe and host.

Gareau *et*. *al*. previously used the Novel Object Recognition and the T-maze tests to demonstrate that short-term recognition and working memory is impaired in female Swiss Webster germ-free mice, regardless of exposure to stress [59]. Our results concur with this study, and extend this finding to include male mice as well. The recognition of novelty requires more cognitive skills, relative to tasks measuring exploration of novel environments or a single novel object [60]. The ability of early *Bifidobacterium* colonization to significantly improve this behavior from what is observed in germ-free mice suggests that the presence of certain bacterial species early in life may contribute to proper cognitive development. The involvement of the hippocampus in short term recognition memory is implicated here and in other studies [31, 59]. Supporting this hypothesis, prior studies have documented molecular and neurochemical changes associated with the hippocampus in germ-free versus SPF mice [11, 12, 59, 61]. That *Bifidobacterium* colonization improves performance on some behavioral tests but not others may indicate that certain brain regions are more susceptible to *Bifidobacterium*-induced modulation than others. These results may provide a key to unravelling the mechanisms of microbe-host communication in future studies.

Our data also indicate that germ-free mice, females in particular, display increased locomotor activity, a result which is in accordance with several previous studies [10, 12]. No previous study has used the Rotarod apparatus to determine whether germ-free mice display an aberrant motor performance phenotype. Our study demonstrates that male germ-free mice display decreased motor performance, characterized both by an initial detriment in coordination and an inability to reach maximum speeds during the test. Both conventionalization and colonization with *Bifidobacterium* spp. improved the motor performance of males in our studies. Motor coordination in rodents is considered to be a measure of cerebellar functionality. The cerebellum is traditionally associated with motor coordination, but has extensive connections with the prefrontal and posterior parietal cortex, and is being increasingly recognized as having a role in emotional, cognitive, and social domains [62]. Moreover, the protracted postnatal development of the cerebellum overlaps in time with the period of rapid microbial colonization of the gut and Diaz Heijtz *et*. *al*. has previously demonstrated that there are more genes affected by germ-free status in the cerebellum than in any other brain region [12]. We therefore suggest that that the cerebellum also be considered as a brain region modulated by the intestinal microbiota, and that bacterial-induced variations in postnatal cerebellar circuit development may result in subtle though biologically meaningful effects on motor performance later in life. The cerebellum’s unique protracted development, sexual dimorphism, and vulnerability to environmental influences make this region a prime target for future studies of the microbiota-gut-brain axis [60].

Several independent groups have tested the sociability phenotype of germ-free Swiss Webster mice using the three-chamber task, though conflicting results have been reported. Arentsen *et*. *al*. reported that germ-free Swiss Webster mice (adult males) displayed higher levels of sociability, while Desbonnet *et*. *al*. reported robust social deficits in germ free mice relative to mice with a complex microbiota [10, 14]. Despite these conflicting results, it can be concluded that the microbiota alters the development of social behavior. In our studies, male germ-free mice spent a nearly equal amount of time engaging in social behavior as the conventionalized and *Bifidobacterium*-colonized mice. This finding may be interpreted to mean that early microbiota colonization does not affect long-term social behavior in males. However, one potential confounding variable in this study was that the sociability testing occurred a week after transfer from the gnotobiotic isolators into the conventional mouse housing facility. Despite being housed in sterile cages and being provided sterile food and water, the mice that were germ-free at the beginning of the week were colonized by environmental microbes by the end of the week. The possibility exists that this short-term colonization was enough to normalize any sociability deficit that would have been apparent in the germ-free males immediately after removal from the gnotobiotic isolator. Conversely, significant differences in sociability based on colonization status were observed in females. Postnatal conventionalization of female Swiss Webster mice resulted in typical social behavior as adults, although the *Bifidobacterium* treatment itself could not rescue the sociability deficit of germ-free females. Arentsen *et*. *al*. has made several observations that may explain the contradictory results, including age at testing and choice of partner mouse genetic background [10]. In addition to these variables, age at colonization and environmental conditions (i.e. housing in identical isolator conditions prior to testing and amount of handling by humans) are also predicted to affect the social behavior of the adult mice. All three treatment groups of mice used in this testing were raised to adulthood in separate but identical gnotobiotic isolators and were handled in the same manner in order to mitigate behavior differences that may have arisen from differential environmental inputs.

The precise mechanisms of microbe-host communication are yet to be fully elucidated [57, 63]. Known mechanisms of microbiome-gut-brain communication include immune system activation, activation of vagal afferents, and signaling through the actions of bacterial products or metabolites. These pathways can either directly or indirectly affect brain function and maturation of brain circuits during critical windows of neurodevelopment. Microbial communities have also been shown to have a dramatic influence on host neurohormones and expression of their cognate receptors which may modulate behavior [64, 65]. Other neuroactive microbial metabolites such as γ-aminobutyric acid (GABA) and histamine may also modulate signaling within the enteric or central nervous systems [29]. Several mechanisms may explain the effects of *Bifidobacterium* species in particular, including stimulation of systemic and intestinal immunity, or production of short-chain fatty acids (SCFAs) such as acetate [66–69]. The increased production of acetate by a *Bifidobacterium*-predominant community may mediate communication between gut microbes and the host CNS by modulating microglia maturation, gene expression, or neurotransmitter function in the developing brain.

Adult behavioral phenotypes are influenced by both host genetics and environmental influences throughout life. The gut microbiota may be an important extrinsic force that shapes host behavior. Multiple lineages of the family Bifidobacteriaceae have been passed down through the generations [70] and have co-evolved with hominids in ways that are beneficial to their hosts. Our microbiota may have lasting effects on neural development and brain function. A *Bifidobacterium*-predominated microbiota in early life is predicted to engage in a complex interaction with the host that has far-reaching effects on adult behavior. With enhanced knowledge of how this beneficial genus affects the CNS in early life, novel therapeutic strategies, such as introduction of select *Bifidobacterium* species to the infant diet, may be designed to exploit these species and elicit desired effects on the brain.

## Supporting information

S1 Table Summary of statistical analyses for all behavioral tests. (PDF)

S1 Extended Methods Additional information regarding gnotobiotic mouse models, germ-free colonization, animal handling, and behavior testing controls. (PDF)

S1 Fig Analysis of beta diversity and taxonomic distribution by sex and treatment group. (PDF)

S2 Fig Longitudinal analysis of taxonomic distribution in conventionalized mice and comparison to donor fecal preparation. (PDF)

S3 Fig *Bifidobacterium* relative abundance and OTU distribution by timepoint and treatment group. (PDF)
