# Supplementary material for: Postnatal colonization with human "infant-type" Bifidobacterium species alters behavior of adult gnotobiotic mice
Source: PLoS One. 2018 May 15;13(5):e0196510. doi: 10.1371/journal.pone.0196510 (PMC5953436; doi:10.1371/journal.pone.0196510)
Supplement: S1 Table — (PDF) [file pone.0196510.s001.pdf]

Supporting Information S1 Table

Luk et. al. (2018) Postnatal colonization with human “infant-type” Bifidobacterium species alters behavior of adult gnotobiotic mice

Behavior domain	Behavior Paradigm	Measurement	Statistical test	Comparison	Statistics	Degrees of freedom	p value	Post hoc Test	Significant comparisons	p value	Figure
Anxiety-like response	Elevated plus maze	Time spent in the open arm (sec)	Two-way ANOVA	Factor 1: Treatment	F=5.38	DFn=2, DFd=50	***0.0077***	Bonferroni MCT	Female: GF vs. CONV	<0.01	3A
				Factor 2: Sex	F=3.92	DFn=1, DFd=50	0.0531				
				Interaction (F1 X F2)	F=2.95	DFn=2, DFd=50	0.0616				
			Kruskal-Wallis	Treatment: Sexes Combined	H=12.34	DFn=2, DFd=25	***0.0021***	Dunns MCT	GF vs. BIF	< 0.05	
									GF vs. CONV	<0.01	
		Time spent in the closed arm (sec)	Two-way ANOVA	Factor 1: Treatment	F=8.27	DFn=2, DFd=50	***0.0008***	Significant interaction between sex and treatment observed. Therefore, *p* values of *post hoc* tests were analyzed separately by sex			3B
				Factor 2: Sex	F=7.15,	DFn=1, DFd=50	***0.0101***				
				Interaction (F1 X F2)	F=8.11	DFn=2, DFd=50	***0.0009***				
			One-Way ANOVA	Treatment: Males	F=4.005	DFn=2, DFd=25	***0.031***	Bonferroni MCT	BIF vs. CONV	< 0.05	
			One-Way ANOVA	Treatment: Females	F=18.03	DFn=2, DFd=25	***<0.0001***	Bonferroni MCT	GF vs. BIF	<0.05	
									GF vs. CONV	<0.0001	
		Distance travelled on open arm (cm)	Two-way ANOVA	Factor 1: Treatment	F=3.78	DFn=2, DFd=50	***0.0295***	Bonferroni MCT	Female: GF vs. CONV	<0.05	3C
				Factor 2: Sex	F=3.23	DFn=1, DFd=50	0.0782				
				Interaction (F1 X F2)	F=1.67	DFn=2, DFd=50	0.1983				
			Kruskal-Wallis	Treatment: Sexes Combined	H=14.97	DFn=2, DFd=25	***0.0006***	Dunns MCT	GF vs. BIF	< 0.05	
									GF vs. CONV	< 0.001	
		Number of entries to open arms	Two-way ANOVA	Factor 1: Treatment	F=8.88	DFn=2, DFd=50	***0.0005***	Significant interaction between sex and treatment observed. Therefore, *p* values of *post hoc* tests were analyzed separately by sex			3D
				Factor 2: Sex	F=3.73	DFn=1, DFd=50	0.0592				
				Interaction (F1 X F2)	F=3.72	DFn=2, DFd=50	***0.0311***				
			Kruskal-Wallis	Treatment: Males	H=4.341	DFn=2, DFd=25	0.1141	Dunns MCT	-	ns	
			One-Way ANOVA	Treatment: Females	F=9.047	DFn=2, DFd=25	***0.0011***	Bonferroni MCT	GF vs. BIF	< 0.05	
									GF vs. CONV	< 0.01	
Locomotor Activity	Open field activity	Total distance travelled (cm)	Two-way ANOVA	Factor 1: Treatment	F=1.99	DFn=2, DFd=50	0.1472	Significant interaction between sex and treatment observed. Therefore, *p* values of *post hoc* tests were analyzed separately by sex			4A
				Factor 2: Sex	F=17.11	DFn=1, DFd=50	***<0.0001***				
				Interaction (F1 X F2)	F=10.64	DFn=2, DFd=50	***<0.0001***				
			One-way ANOVA	Treatment: Males	F=3.085	DFn=2, DFd=25	0.0635	Bonferroni MCT	-	ns	
			One-way ANOVA	Treatment: Females	F=8.726	DFn=2, DFd=25	***0.0013***	Bonferroni MCT	GF vs. CONV	< 0.01	
									CONV vs. BIF	< 0.01	
		Movement time	Two-way ANOVA	Factor 1: Treatment	F=1.72	DFn=2, DFd=50	0.1903	Significant interaction between sex and treatment observed. Therefore, *p* values of *post hoc* tests were analyzed separately by sex			4B
				Factor 2: Sex	F=12.80	DFn=1, DFd=50	***0.0008***				
				Interaction (F1 X F2)	F=10.37	DFn=2, DFd=50	***0.0002***				
			One-way ANOVA	Treatment: Males	F=5.276	DFn=2, DFd=25	***0.0123***	Bonferroni MCT	CONV vs. BIF	< 0.05	
			One-way ANOVA	Treatment: Females	F=7.095	DFn=2, DFd=25	***0.0036***	Bonferroni MCT	GF vs. CONV	< 0.05	
									CONV vs. BIF	< 0.01	
		Number of rears	Two-way ANOVA	Factor 1: Treatment	F=5.36	DFn=2, DFd=50	***0.0078***	Significant interaction between sex and treatment observed. Therefore, *p* values of *post hoc* tests were analyzed separately by sex			4C
				Factor 2: Sex	F=11.31	DFn=1, DFd=50	***0.0015***				
				Interaction (F1 X F2)	F=12.52	DFn=2, DFd=50	***<0.0001***				
			One-way ANOVA	Treatment: Males	F=2.340	DFn=2, DFd=25	0.1171	Bonferroni MCT	-	ns	
			One-way ANOVA	Treatment: Females	F=16.06	DFn=2, DFd=25	***<0.0001***	Bonferroni MCT	GF vs. CONV	< 0.0001	
									CONV vs. BIF	< 0.001	
		Time spent rearing	Two-way ANOVA	Factor 1: Treatment	F=4.17	DFn=2, DFd=50	***0.0211***	Significant interaction between sex and treatment observed. Therefore, *p* values of *post hoc* tests were analyzed separately by sex			4D
				Factor 2: Sex	F=4.74	DFn=1, DFd=50	***0.0342***				
				Interaction (F1 X F2)	F=9.27	DFn=2, DFd=50	***0.0004***				
			One-way ANOVA	Treatment: Males	F=2.570	DFn=2, DFd=25	0.0966	Bonferroni MCT	-	ns	
			One-way ANOVA	Treatment: Females	F=11.89	DFn=2, DFd=25	***0.0002***	Bonferroni MCT	GF vs. CONV	<0.001	
									CONV vs. BIF	<0.05	

S1 Table. Summary of statistical analyses for all behavioral tests

GF=Germ-free (n=9m/13f)

CONV= Conventionalized (n=11m/8f)

BIF = *Bifidobacterium*-treated (n=8m/9f)

Supporting Information S1 Table

Luk et. al. (2018) Postnatal colonization with human “infant-type” Bifidobacterium species alters behavior of adult gnotobiotic mice

Behavior domain	Behavior Paradigm	Measurement	Statistical test	Comparison	Statistics	Degrees of freedom	p value	Post hoc Test	Significant comparisons	p value	Figure
Motor Coordination/ Performance	RotaRod	Latency to Fall	Two-way Repeated Measures ANOVA	Treatment: Males	F=2.89	DFn=2, DFd=25	0.0742	Bonferroni MCT	Trial 1: GF vs. CONV	<0.05	5A
									Trial 3: GF vs. CONV	<0.05	
			Two-way Repeated Measures ANOVA	Treatment: Females	F=2.96	DFn=2, DFd=25	0.0700	Bonferroni MCT	Trial 1: GF vs. BIF	<0.05	
		Average Latency to Fall (across all trials)	Two-way ANOVA	Factor 1: Treatment	F=5.04	DFn=2, DFd=50	***0.0101***	Bonferroni MCT	-	ns	5B
				Factor 2: Sex	F=2.60	DFn=1, DFd=50	0.1128				
				Interaction (F1 X F2)	F=0.94	DFn=2, DFd=50	0.23988				
			Kruskal-Wallis	Treatment: Sexes Combined	H=7.867	DFn=2, DFd=25	***0.0196***	Dunns MCT	GF vs. BIF	<0.05	
		Weight	One-way ANOVA	Treatment: Males	F=3.731	DFn=2, DFd=25	***0.0382***	Bonferroni MCT	-	ns	5E
			One-way ANOVA	Treatment: Females	F=2.081	DFn=2, DFd=25	0.1498	Bonferroni MCT	-	ns	
Learning and Memory	Novel Object Recognition	Recognition Index	Two-way ANOVA	Factor 1: Treatment	F=57.46	DFn=2, DFd=49	***<0.0001***	Bonferroni MCT	Females: GF vs. BIF	<0.0001	6B
									Females: GF vs. CONV	<0.0001	
				Factor 2: Sex	F=0.11	DFn=1, DFd=49	0.7369		Males: CONV vs. BIF	<0.01	
				Interaction (F1 X F2)	F=2.16	DFn=2, DFd=49	0.1266		Males: GF vs. CONV	<0.0001	
			One-Way ANOVA	Treatment: Sexes Combined	F=61.58	DFn=2, DFd=52	***<0.0001***	Bonferroni MCT	GF vs. CONV	<0.0001	
									GF vs. BIF	<0.0001	
									CONV vs. BIF	<0.001	
Social Behavior	Sociability	Interaction time: Males	Two-way ANOVA	Factor 1: Social choice	F=98.12	DFn=1, DFd=50	***<0.0001***	Bonferroni MCT	GF: Mouse vs. Object	<0.0001	7A
				Factor 2: Treatment	F=1.96	DFn=2, DFd=50	0.1513		CONV: Mouse vs. Object	<0.0001	
				Interaction (F1 X F2)	F=0.54	DFn=2, DFd=50	0.5872		BIF: Mouse vs. Object	<0.001	
		Interaction time: Females	Two-way ANOVA	Factor 1: Social choice	F=23.72	DFn=1, DFd=50	***<0.0001***	Bonferroni MCT	GF: Mouse vs. Object	ns	
				Factor 2: Treatment	F=3.06	DFn=2, DFd=50	0.0557		CONV: Mouse vs. Object	<0.0001	
				Interaction (F1 X F2)	F=5.48	DFn=2, DFd=50	***0.0070***		BIF: Mouse vs. Object	ns	
		Sociability Index	Two-way ANOVA	Factor 1: Treatment	F=5.30	DFn=2, DFd=50	***0.0082***	Significant interaction between sex and treatment observed. Therefore, *p* values of *post hoc* tests were analyzed separately by sex			7B
				Factor 2: Sex	F=8.65	DFn=1, DFd=50	***0.0049***				
				Interaction (F1 X F2)	F=5.02	DFn=2, DFd=50	***0.0103***				
			One-Way ANOVA	Treatment: Males	F=0.001	DFn=2, DFd=50	0.9863	Bonferroni MCT	-	ns	
			Kruskal-Wallis	Treatment: Females	H=13.44	DFn=2, DFd=50	***0.0012***	Dunn's MCT	GF vs. CONV	<0.01	
									BIF vs. CONV	<0.01	

S1 Table. Summary of statistical analyses for all behavioral tests

GF=Germ-free (n=9m/13f)

CONV= Conventionalized (n=11m/8f)

BIF = *Bifidobacterium*-treated (n=8m/9f)
