# Supplementary material for: Postnatal colonization with human "infant-type" Bifidobacterium species alters behavior of adult gnotobiotic mice
Source: PLoS One. 2018 May 15;13(5):e0196510. doi: 10.1371/journal.pone.0196510 (PMC5953436; doi:10.1371/journal.pone.0196510)
Supplement: S1 Extended Methods — (PDF) [file pone.0196510.s002.pdf]

# Supporting Information S1 Extended Methods

This supplemental information includes additional experimental information regarding gnotobiotic mouse models, germ-free colonization, animal handling, and behavior testing controls.

**Gnotobiotic mouse models** Germ-free Swiss Webster Mice (outbred) were purchased from Taconic, and bred in the germ-free facility at Baylor College of Medicine to create an outbred colony of germ-free mice. Previous behavior studies have utilized Swiss Webster mice, making this strain useful for study comparisons and attractive as an outbred mouse model. The large litter size and superior nurturing ability of the Swiss Webster dam also made this mouse model ideal, as the manipulations with the pups puts them at risk of cannibalism in many other strains of mouse. At approximately 6-8 weeks of age, timed pregnancies were induced in adult female mice in the germ-free colony isolators. Vaginal plugs were used to identify pregnant dams, which were moved to separate experimental isolators. The day of birth was considered as P0. Pregnant dams and their litters were maintained under germ-free or gnotobiotic conditions in separate flexible isolators fed with HEPA-filtered air. Mice were kept under filter-top cages with *ad libitum* access to irradiated food and water. All animals were housed under 12 hour light – 12 hour dark cycle. Animal care and experimental procedures were approved by the Institutional Animal Care and Use Committee (IACUC) at Baylor College of Medicine, Houston, TX, in accordance with all guidelines set forth by the U.S. National Institutes of Health (Baylor IACUC approved protocols #AN-6530 and #AN-6706).

**Colonization of germ-free mice** The *Bifidobacterium* strains were grown in culture conditions described above. Overnight bacterial cultures were combined in equal ratios based on $OD_{600}$ readings ($OD_{600}$ of only the dams were given the gavage of fecal matter (At P1), and used bedding material from the cage of SPF donor mice was added to the cage with the dam and new pups (on P1 and P3). ~1.0/species), pelleted, and re-suspended in sterile phosphate buffered saline (Gibco, Grand Island, NY, USA). This mixture of the four strains of *Bifidobacterium* used for the gavages is referred to throughout the text as the "*Bifidobacterium* treatment". The dam was administered 0.2 mL of this treatment via oral gavage every other day post-partum. The mixture was also administered to the drinking water to increase exposure and likelihood of colonization. Pups were treated with 0.02 mL of the gavage mixture starting at P1, and every other day thereafter until P20. This persistent treatment ensured that all four human *Bifidobacterium* species were present during this critical developmental window, even if they were not able to readily colonize the murine intestine in the absence of human milk oligosaccharides. Pups between the ages of P1-P10 were not orally gavaged, rather the mixture was administered slowly to their mouths via pipette tip. From P10 onwards, the gavage treatment was administered with an 18 gauge ball-tipped gavage needle. Dilution plating of the gavage mixture at several points during the experiment on MRS plates confirmed that the dams received approximately $1.1x10^{10}$ CFUs (colony forming units – i.e. viable bacterial cells) per gavage, and the pups received approximately $1.1x10^{9}$ CFUs. The pups were then weaned at P21 (2-5 mice per cage), and received a single gavage of the *Bifidobacterium* treatment once per week until behavior testing (13 total treatments).

Control germ-free mice received gavages of sterile PBS in the same manner as described above. Germ-free status was monitored over the course of the experiment by plating fresh fecal pellets from the dams on blood agar plates (Hardy Diagnostics, Santa Monica, CA, USA) and incubating both aerobically and anaerobically at 37°C. An absence of bacterial colonies on these plates 48 hours later was considered negative for bacterial contamination.

Specific Pathogen Free (SPF) mice are housed in traditional mouse housing facilities and have what is considered a healthy gut microbiota that colonizes naturally from exposure to the dam, other mice, and the environment in early life. Conventionalized (CONV) litters were colonized by administering a fecal matter preparation derived from SPF donor mice (strain, age, and sex-matched) to the dams via gavage and by adding fecal matter from the SPF donor cage to the cage containing the dam and litter of pups. A single cage of SPF, Swiss Webster, female, adult (6 weeks old) mice were used as the feces donors, in order to maintain consistency throughout the experiment (length of study was based on differential birth dates of the litters).  To colonize the dam, 3-4 fecal pellets were vortexed rigorously in sterile PBS, then centrifuged at 3000 x g for 5 minutes. The resulting supernatant was used as the SPF fecal matter preparation and was administered to the dams via gavage on P1. The conventionalized pups however were not directly given a gavage of this adult fecal matter preparation, as that would not represent a natural form of colonization (as it would be a forced presence of “adult-type” bacterial species) and would not accurately model the true biological situation (gradual colonization based on contact with the dam and environment). Instead, only the dams were given the gavage of fecal matter (At P1), and used bedding material from the cage

of SPF donor mice was added to the cage with the dam and new pups (on P1 and P3). In this way, the pups were colonized via close interaction with the dam, and via environmental exposure to the fecal matter in the bedding. Therefore, whichever microbial species initially colonized the guts of the pups (rather than pass through transiently) did so through a natural route. Agar plating of intestinal content from these pups on blood agar plates confirmed that this method was sufficient to colonize the pups in these cages by P4. In order to maintain consistent handling throughout development between the groups, the conventionalized pups were also administered a gavage of sterile PBS in the same manner as described for the germ-free cohort.

**Mouse handling and behavior testing** Offspring in each treatment group were raised to adulthood in identical gnotobiotic isolators and were handled in the same manner in order to mitigate behavior differences that may arise from these environmental inputs. At 6-7 weeks of age, the mice were then transferred into sterile cages and moved to the facility housing the behavioral testing equipment. The mice were allowed to acclimate to the new environment for 24 hours after removal from the gnotobiotic isolators, after which behavioral testing commenced. We examined one assay per behavior domain, and limited the test battery to one week in order to minimize exposure to environmental sources of bacterial contamination to the best of our ability. Tests were performed from the least to the most invasive to minimize the influence of prior test history. However, the social interaction test was performed last so as to minimize exposure to the microbiota of partner mice used in this test. The mice were housed in sterile cages and provided sterile food and water during the behavior testing. Handling was minimized, and all equipment was cleaned thoroughly with 70% ethanol between

tests. All tests were run under dimmed lighting (70 lux) with white noise background (60 dB) to avoid distraction and minimize anxiety. All testing occurred between 1200-1900 hours and mice were allowed to habituate to the testing environment for 30 minutes prior to testing. Mice were placed in holding cages post-test to avoid interactions with untested animals. All animal cages were returned to the housing room between test days and animals were sacrificed 24-48 hours after the last test. The 3 cohorts of mice were tested during different months based on gnotobiotic isolator availability (October, December, and March). In order to account for this, 2 male and 2 female, age-matched, germ-free Swiss Webster mice were also tested along with each group in order to ensure no seasonal effects skewed results of testing. After behavioral testing, mice were euthanized either by $CO_2$ inhalation under controlled conditions via a Euthanex chamber or in cases where tissue was collected, cervical disarticulation under surgical plane of anesthesia. As required by institutional policies, secondary methods to ensure death were utilized including one of the aforementioned methods, in addition to decapitation or bilateral opening of the thorax. All euthanization procedures were performed by research personnel approved on the Baylor College of Medicine IACUC protocol #AN-6706.
