# Supplementary material for: Postnatal colonization with human "infant-type" Bifidobacterium species alters behavior of adult gnotobiotic mice
Source: PLoS One. 2018 May 15;13(5):e0196510. doi: 10.1371/journal.pone.0196510 (PMC5953436; doi:10.1371/journal.pone.0196510)
Supplement: S1 Fig — (PDF) [file pone.0196510.s003.pdf]

Supporting Information S1 Fig

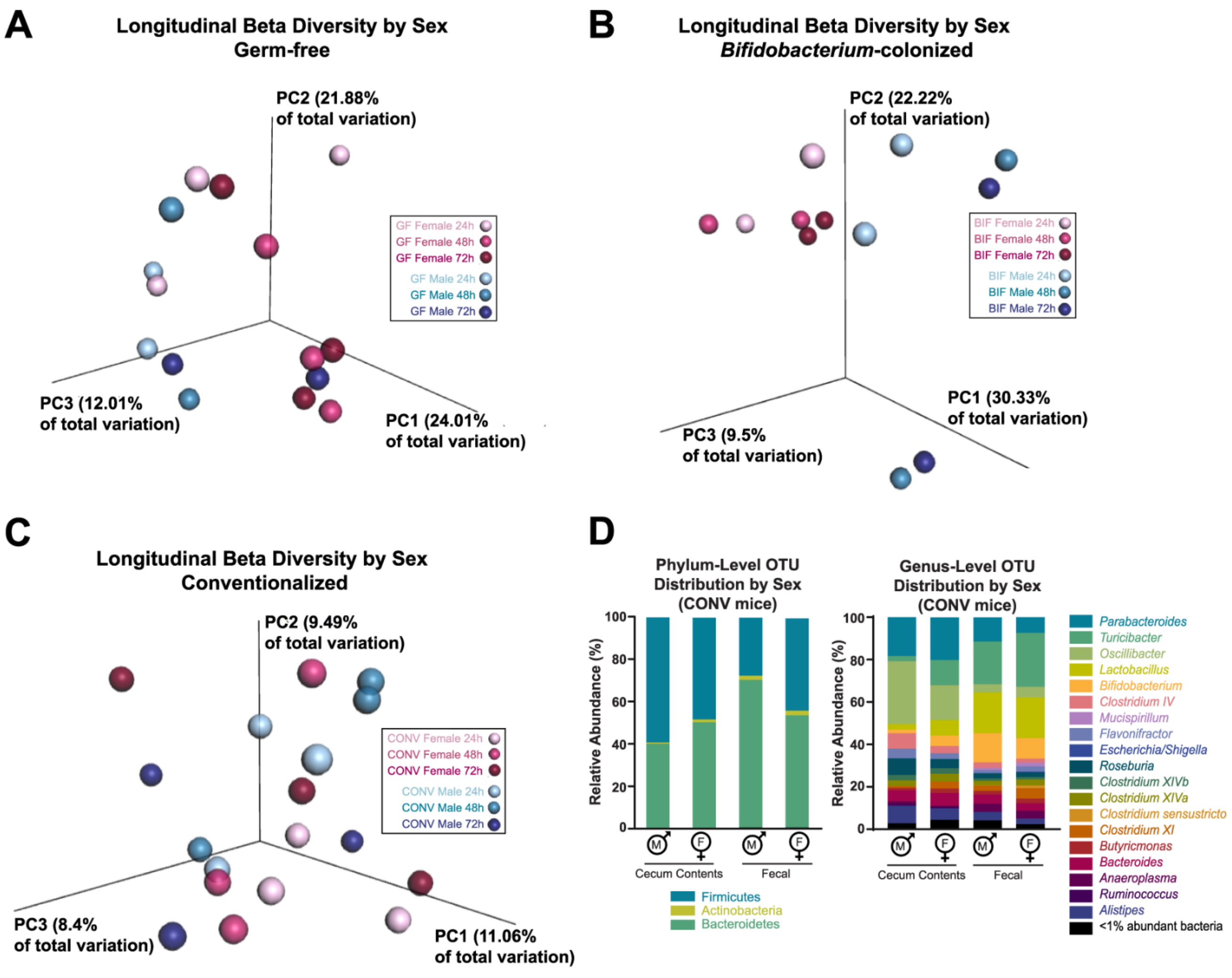

Supporting Information S1 Fig. Analysis of Beta Diversity and taxonomic distribution by sex and treatment group.

(A-C) Principal Coordinates Analyses (PCoA) for **(A)** Germ-free, **(B)** *Bifidobacterium*-colonized, and **(C)** Conventionalized cohorts over the first 72 hours post-transfer from gnotobiotic isolators. The percentage variation explained by each of the three primary principal factors is indicated on each axis. Coordinates representing individual samples are colored according to group, with distance to other coordinates indicating similarity/dissimilarity. **(D)** Relative abundance of operational taxonomic units (OTUs) in the CONV group of mice distributed by sex and location of sampling. Left panel indicates phylum–level comparison and right panel denotes genus-level comparisons. (fecal microbiome analysis: n=3m/3f per timepoint totaling n=9m/9f per treatment; cecum content microbiome analysis n=5m/5f BIF, 6m/6f GF, and 9m/5f CONV) GF = germ-free; CONV = conventionalized; BIF =*Bifidobacterium*-colonized
