# Supplementary material for: Postnatal colonization with human "infant-type" Bifidobacterium species alters behavior of adult gnotobiotic mice
Source: PLoS One. 2018 May 15;13(5):e0196510. doi: 10.1371/journal.pone.0196510 (PMC5953436; doi:10.1371/journal.pone.0196510)
Supplement: S2 Fig — (PDF) [file pone.0196510.s004.pdf]

Supporting Information S2 Fig

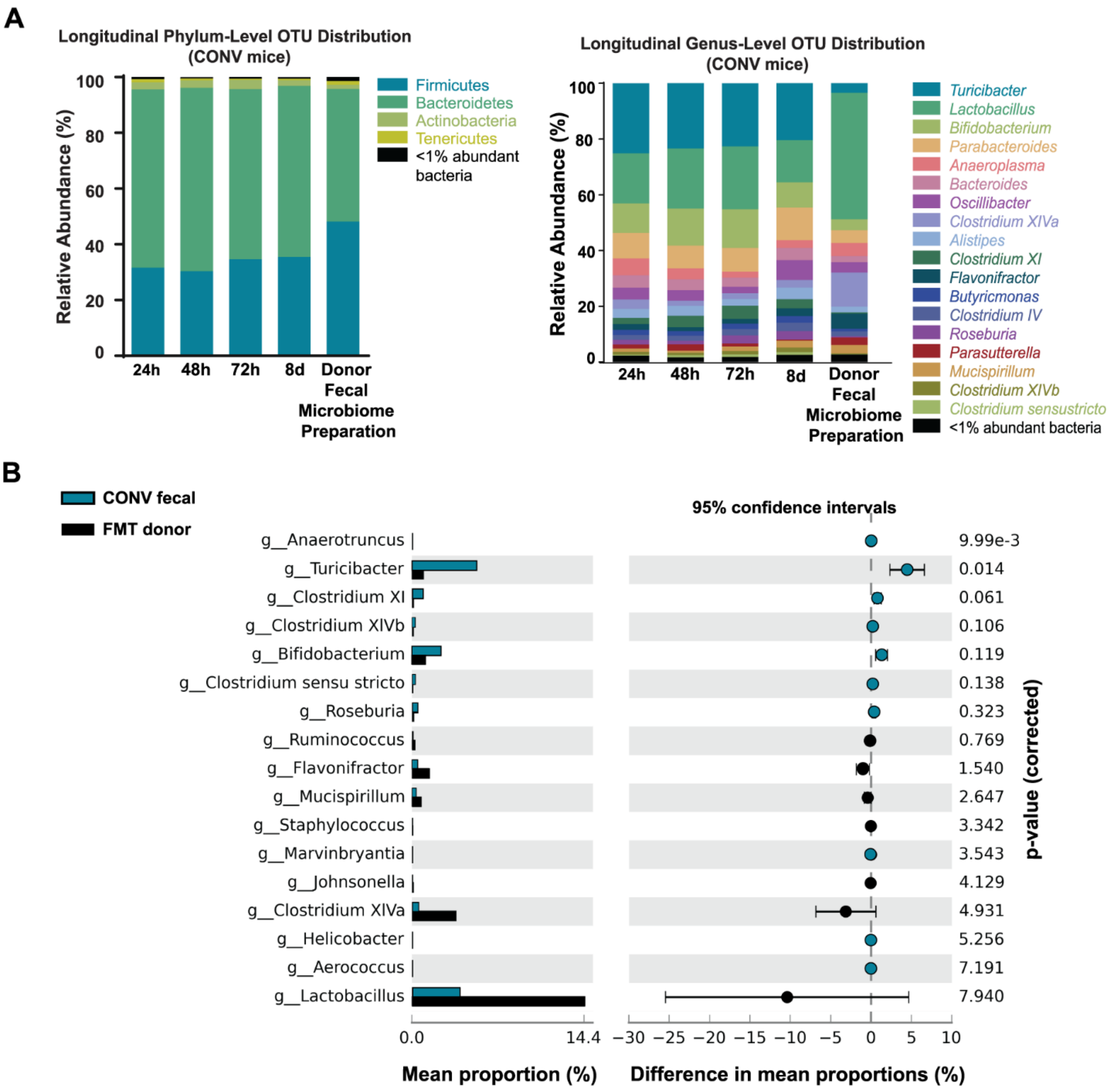

S2 Fig. Longitudinal analysis of taxonomic distribution in conventionalized mice and comparison to donor fecal preparation.

(A) Relative abundance of operational taxonomic units (OTUs) in fecal microbiome profiles from conventionalized mice expanded to include last sampling, 8 days after transfer. Plot also compares composition to SPF donors. Left panel indicates the phylum–level comparison and right panel denotes genus-level comparisons. **(B)** Extended bar plots showing differences in mean proportions (%) at genus level between the conventionalized fecal microbiome profile and the FMT donor microbiome profile. Corrected p-value for each comparison shown at right of plots (n=3m/3f per timepoint totaling n=9m/9f per treatment). GF = germ-free; CONV = conventionalized; BIF = *Bifidobacterium*-colonized
