# Supplementary material for: Postnatal colonization with human "infant-type" Bifidobacterium species alters behavior of adult gnotobiotic mice
Source: PLoS One. 2018 May 15;13(5):e0196510. doi: 10.1371/journal.pone.0196510 (PMC5953436; doi:10.1371/journal.pone.0196510)
Supplement: S3 Fig — (PDF) [file pone.0196510.s005.pdf]

Supporting Information S3 Fig

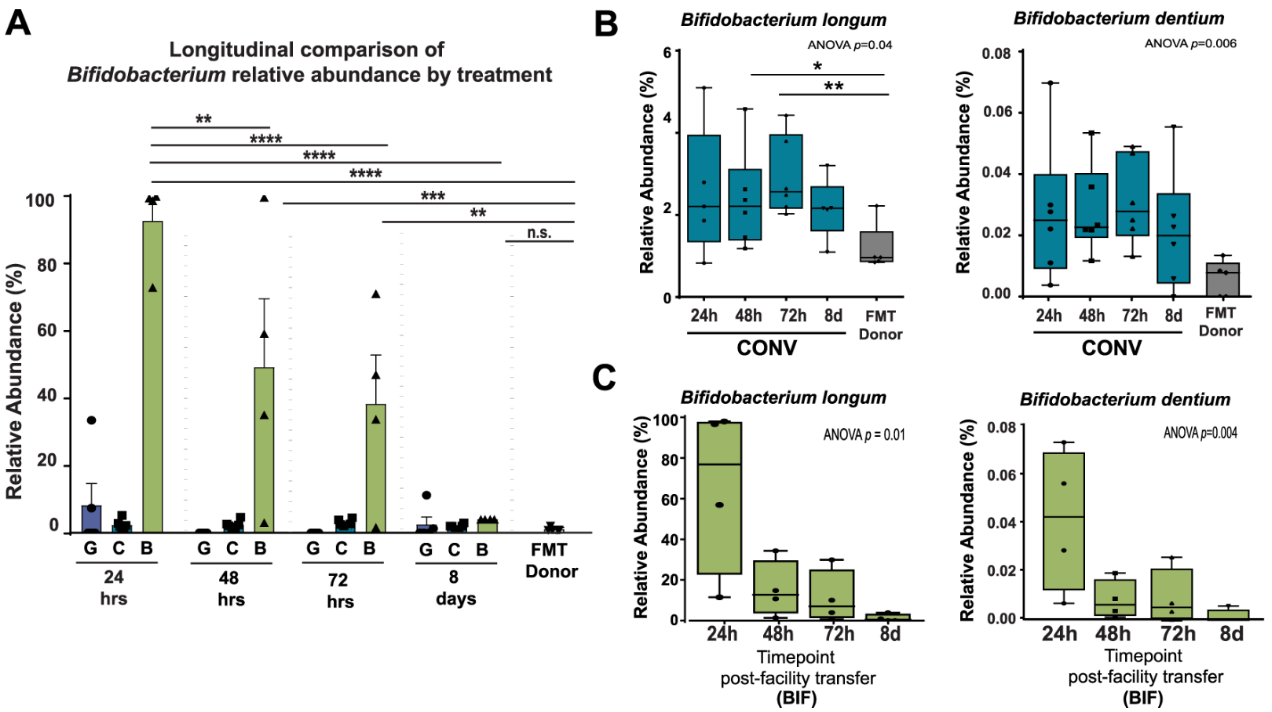

Supporting Information S3 Fig. *Bifidobacterium* relative abundance and OTU distribution by timepoint and treatment group

(A) Relative abundance of *Bifidobacterium* Operational Taxonomic Units (OTUs) in the fecal microbiome profiles of the fecal microbiome transplant (FMT) donors and the germ-free, conventionalized, and *Bifidobacterium*-colonized cohorts. Plots represent data at 24, 48, and 72 hours after transfer from the gnotobiotic isolators, and on day 8 (last day of study). **(B-C)** Species-level OTUs identified as *Bifidobacterium* (*Bifidobacterium longum* and *Bifidobacterium dentium*) in the conventional and FMT donor microbiome profiles (B) and the *Bifidobacterium*-colonized group (C) are plotted for each timepoint after transfer from the gnotobiotic isolators. All data are presented as means ±SEM (n = 4-6 mice per timepoint/treatment group). *p < 0.05, **p<0.01, ***p<0.001, ****p<0.0001, n.s.=not significant (germ-free=GF=G, Conventionalized=CONV=C, and *Bifidobacterium*-colonized=BIF=B)
